# A transferable double exponential potential for condensed phase simulations of small molecules†

**DOI:** 10.1039/d3dd00070b

**Published:** 2023-07-10

**Authors:** Joshua T. Horton, Simon Boothroyd, Pavan Kumar Behara, David L. Mobley, Daniel J. Cole

**Affiliations:** a School of Natural and Environmental Sciences, Newcastle University Newcastle upon Tyne NE1 7RU UK daniel.cole@ncl.ac.uk; b Boothroyd Scientific Consulting Ltd London WC2H 9JQ UK; c Department of Pharmaceutical Sciences, University of California Irvine California 92697 USA; d Department of Chemistry, University of California Irvine California 92697 USA

## Abstract

The Lennard–Jones potential is the most widely-used function for the description of non-bonded interactions in transferable force fields for the condensed phase. This is not because it has an optimal functional form, but rather it is a legacy resulting from when computational expense was a major consideration and this potential was particularly convenient numerically. At present, it persists because the effort that would be required to re-write molecular modelling software and train new force fields has, until now, been prohibitive. Here, we present Smirnoff-plugins as a flexible framework to extend the Open Force Field software stack to allow custom force field functional forms. We deploy Smirnoff-plugins with the automated Open Force Field infrastructure to train a transferable, small molecule force field based on the recently-proposed double exponential functional form, on over 1000 experimental condensed phase properties. Extensive testing of the resulting force field shows improvements in transfer free energies, with acceptable conformational energetics, run times and convergence properties compared to state-of-the-art Lennard–Jones based force fields.

## Abstract 2

The Open Force Field software stack is employed to automatically train a transferable, small molecule force field, based on the double exponential functional form, on over 1000 experimental condensed phase physical properties.

## Graphical abstract

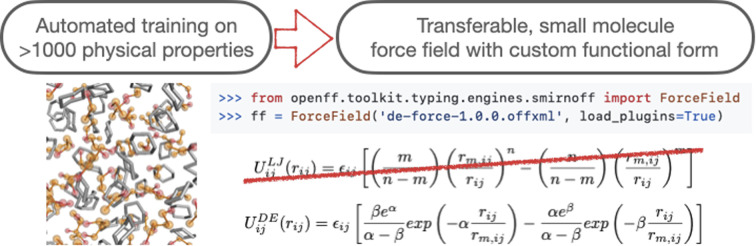

## 1 Introduction

Classical molecular mechanics force fields are widely used for the modelling of molecules and materials, particularly in computational chemistry and biology where the length and time scales of interest would be prohibitive for other more rigorous methods. For all of the fixed charge small molecule and biological families of force fields in common use today,^1^ non-bonded interactions between atoms are treated by the sum of a Coulomb term and a Lennard–Jones (LJ) potential given by:^2,3^1
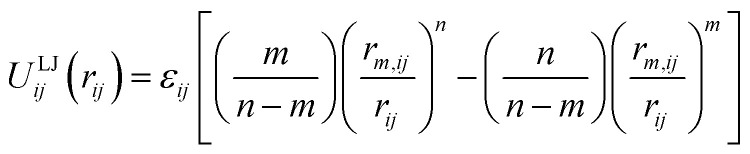
Here, atoms *i* and *j* have two tunable parameters, the equilibrium separation *r*_*m*,*ij*_ and well depth *ε*_*ij*_. The 12–6 potential (*n* = 12, *m* = 6) is almost universally used for computational ease, simply because the repulsive 12th power can be obtained by squaring the *r*^−6^ term.^2^ The simplicity of the LJ potential makes the parameterisation process more tractable and allows for fast computation, but there are limited physical reasons for its widespread use.^4^ In particular, although the leading order term in the attractive dispersion interaction decays as *r*^−6^, the LJ potential neglects (or must effectively account for) the higher order terms in the expansion.^5–7^ The *r*^−12^ term seeks to model repulsion between electrons, but an exponential decay is more physically justified and provides a more accurate description of repulsion at short distances.^8^ A further technical issue with the LJ potential is its divergence as *r* tends to zero. This is not typically a problem in standard molecular dynamics simulations, but can lead to poor convergence and systematic errors in alchemical free energy calculations as atoms are (dis)appeared. In these cases, the LJ potential must be replaced by a (less simple) soft-core potential,^9^ which makes these calculations significantly more difficult and computationally expensive.

Recently, an alternative to the LJ function has been suggested for condensed phase modelling, named the double exponential (DE) potential:^10,11^2
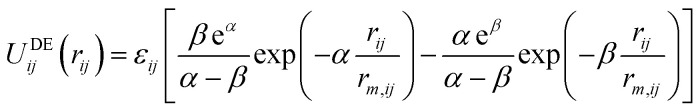
Here, the parameters *ε*_*ij*_ and *r*_*m*,*ij*_ have exactly the same physical interpretation as in the LJ functional form (eqn (1)). The two extra parameters, *α* and *β*, control the steepness of the repulsive interaction and decay of the attractive interaction, respectively, thus offering control over the shape of the potential energy well.^10^ The repulsive term of the DE potential therefore takes on a physically-motivated exponentially decaying form. Meanwhile, the attractive term has additional flexibility, compared to LJ, to effectively account for the induced multipole, such as charge–dipole, charge–quadrupole and dipole-quadrupole, as well as the many body interactions, that contribute to dispersive interactions in the condensed phase.^11^ An additional advantage of the DE potential is its natural soft core, which ensures that the energy is finite at *r* = 0:3
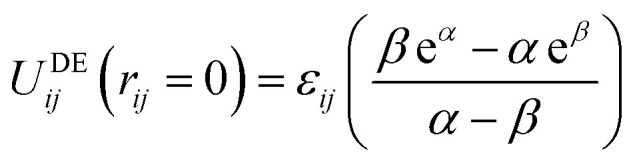
Despite the more complex functional form, similar timings have been reported for implementations of the DE and LJ potentials in a CPU version of the pmemd software.^11,12^ Thus, there is an opportunity to improve the accuracy of condensed phase modelling through extra flexibility in the functional form of the DE potential, and convergence of free energy calculations through its natural soft core, at acceptable computational cost.

An early application of the DE potential has been to the hydration of divalent metal ions, using a three-point water model (named DE-TIP3P).^11^ Parameter sweeps (for *α*, *β*, *ε* and *r*_*m*_) were performed with the goal of reproducing experimental water density and O–O radial distribution functions, and subsequently hydration free energies of ions in the obtained water model. The resulting DE force field performed better than a commonly-used LJ model,^13^ and comparably to a 12-6-4 nonbonded potential^14^ and an alternative model for metals that includes dummy ions,^15^ thus demonstrating the flexibility of the functional form.

While this early application of the DE potential makes a good proof-of-concept, new infrastructure and more work is needed to produce a full force field. In particular, in order to produce a general, transferable small molecule force field comparable with main-line LJ-based force fields, such as OPLS^16^ or GAFF,^17^ it is desirable to implement robust, automated methods for parameter fitting, over a wide range of experimental properties and chemical space.

### 1.1 Open force field infrastructure automates robust parameter fits

The Open Force Field (OpenFF) Initiative is an academic-industrial partnership aiming to advance the science and software infrastructure required to build the next generation of molecular mechanics force fields. OpenFF software infrastructure includes modern, automated frameworks for force field parameter assignment *via* direct chemical perception.^18^ In contrast to most force fields, where an atom is first assigned an atom type based on its chemical environment, in chemical perception the force field parameters are assigned directly from chemical substructures (defined by SMIRKS patterns). Importantly, this direct assignment significantly reduces the number of force field parameters, and facilitates their rapid and robust training through force field optimisation techniques. The ForceBalance software^19^ is employed for fitting of bonded and non-bonded parameter sets against quantum chemical and/or experimental datasets. OpenFF-Evaluator^20^ provides a highly scalable framework for evaluating physical properties, and their gradients, based on the provided force field parameters. Evaluator interfaces with ForceBalance to enable the training, and at-scale testing, of force field non-bonded parameters against curated condensed phase physical property data, including mixture properties.^21^

The first generations of OpenFF force fields (Parsley^22^ and Sage^23^) to be trained using this infrastructure have been released, and show competitive accuracy when estimating quantum chemical conformational energetics, various physical property measurements, and protein–ligand binding free energies. However, until now, there has not been any departure from the commonly-used LJ-based force field functional forms. Here, we introduce Smirnoff-plugins as a means to support nonbonded potentials, of arbitrary functional forms, within the OpenFF software stack. We use this new architecture to automate the training of a new, transferable, small molecule DE force field parameter library for use in condensed phase simulations, and demonstrate improvements against the Sage force fields across all condensed phase metrics. We further evaluate the DE force field against experimental transfer free energies between aqueous and non-aqueous solvents, which is a promising surrogate for protein–ligand binding, and again demonstrate advantages over the LJ functional form. The final force field is available for use in OpenMM,^24^ including support for GPU acceleration. The parameter library, and instructions for use with the Open Force Field Toolkit, are freely available at https://github.com/jthorton/de-forcefields.

## 2 Results

### 2.1 Smirnoff-plugins enables the training of water models with arbitrary functional forms

**Fig. 1:**
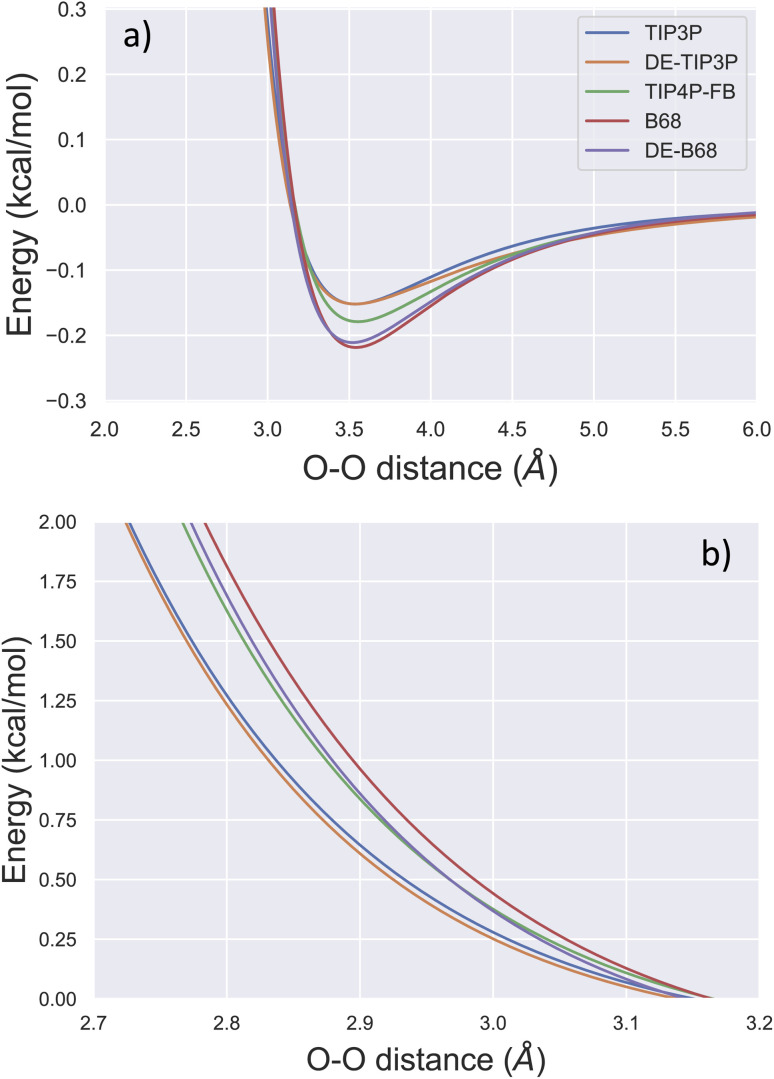
Comparison of O–O non-bonded interactions between two water molecules. The non-bonded component of the interaction energy (excluding electrostatics) is shown for the TIP3P,^25^ DE-TIP3P,^11^ TIP4P-FB,^26^ B68,^27^ and DE-B68 (following training against water condensed phase properties) curves centred on (a) the attractive and (b) the repulsive regions of the interaction potential.

Due to its ubiquity in molecular modelling, there are many water force field models available, including LJ-based force fields with various parameterisations, as well as more sophisticated functional forms.^28^Fig. 1 shows the form of some typical non-bonded interaction potentials between two water oxygen atoms (excluding electrostatics). As discussed, a DE-based three-point water model (DE-TIP3P) has already been developed, by fitting the *α* and *β* parameters of eqn (2) to reproduce the experimental water density and oxygen–oxygen distances at a single temperature.^11^ The resulting DE-TIP3P potential is similar in shape to that of the widely used TIP3P LJ model (Fig. 1).

A number of recent water models have included a much wider range of experimental data in their training, facilitated by automated least-squares optimisation of physical properties with respect to the force field parameters in the ForceBalance software. Fig. 1 shows a LJ-based four point model trained in this way (TIP4P-FB),^26^ as well as a water model that uses a Buckingham-6-8 (B68) non-bonded potential.^27^ The latter model (ESI S1†) has a more physically-motivated functional form than the LJ potential,^4^ but is more difficult to fit (many more tunable parameters) and is computationally expensive (a damping function is required to avoid instability close to *r* = 0).

To illustrate the flexibility of the DE functional form, and the utility of Smirnoff-plugins (Section 4.1) interfaced with the OpenFF software stack, we re-fit a new DE-based four-point water model. The starting parameters for the training run come from a curve fit of the DE *α*, *β*, *ε* and *r*_*m*_ parameters (eqn (2)) to the trained O–O potential parameters from a literature B68 model (Fig. S1†).^27^ We name this model DE-B68 to highlight the origin of the starting parameters and to distinguish it from the earlier DE-TIP3P water model.^11^ Unlike the DE-TIP3P water model, all parameters of the DE-B68 water model, along with the atomic partial charges and O–X distance, were re-optimised automatically using ForceBalance against a wide range of experimental properties. The experimental target training data include the liquid density (*ρ*), heat of vaporization (Δ*H*_vap_), thermal expansion coefficient (*α*), isothermal compressibility (*κ*_T_), isobaric heat capacity (*C*_p_) and the dielectric constant (*ε*(0)) at a range of temperatures and pressures (Section 4.2).

**Fig. 2:**
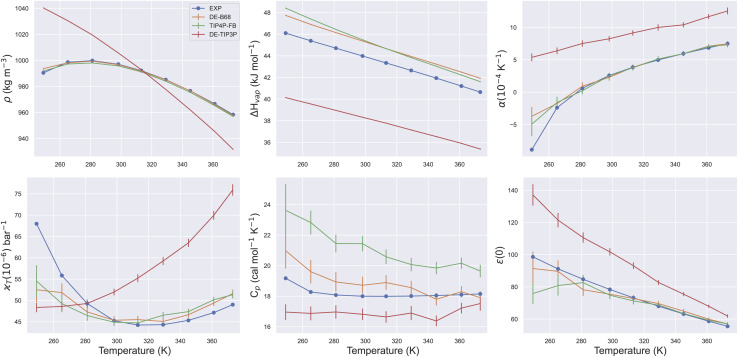
Water physical properties at a range of temperatures. Plots show series of ForceBalance single point property calculations for the DE-B68, DE-TIP3P and TIP4P-FB water models. All properties were used in fitting the DE-B68 and TIP4P-FB force fields, but DE-TIP3P has only been trained at a single temperature. Error bars report the standard deviation and are included for all calculated properties.

The trained DE-B68 function is plotted in Fig. 1, and follows closely the potential energy surface of the more complex B68 function, particularly in the attractive region, despite the former's fewer tunable parameters. The full set of DE-B68 water model parameters is listed in Table S1†. Fig. 2 shows the performance of the published TIP4P-FB^26^ and DE-TIP3P^11^ force fields, as well as the new DE-B68 water model, on a range of physical properties, compared to experiment. The DE-TIP3P model was only fit to water density and oxygen–oxygen distances at a single temperature, and as such does not extrapolate well outside its training regime. On the other hand, the TIP4P-FB and DE-B68 models show similar, strong performance, within the error bars, across the set of properties studied here. Thus, with access to robust, automated parameter fitting, the DE potential shows promise as an alternative to LJ-based potentials in condensed phase modelling, at least in terms of providing viable water models.

### 2.2 A transferable DE-based force field for small molecules

Given this success, we wanted to test DE-based potentials beyond water models. Traditionally, the journey from hypothesis to a general, transferable force field that accurately covers extensive chemical space, such as the OPLS^16^ or GAFF^17^ small molecule force fields, requires decades of development. This may help to explain why the LJ potential has persisted in mainline force fields for so long, despite its known shortcomings; exploring alternatives would have been too expensive and costly for most practitioners.

The Smirnoff-plugins interface with the OpenFF software stack now enables us to generate such a DE-based force field for small, organic molecules automatically and robustly, so we did so here. We started a non-bonded parameter fit from the set of *ε* and *r*_*m*_ for 15 SMIRKS types from the recent LJ-based OpenFF Sage force field which are explicitly exercised by the available training data (Fig. S2†). We note that an additional 20 SMIRKS types, including those covering S, P, I, F and counter-ions, were therefore not optimised in this first study, but can be added in future using the procedures described here. Water parameters and the global *α* and *β* parameters were initialised to their values from the optimised DE-B68 model, but allowed to vary. The Sage condensed phase mixture set (Section 4.2) was used for training, which includes 555 pure and binary densities, and 477 enthalpies of mixing. In addition, pure water densities at a range of temperatures were included as targets to ensure the water model remained accurate. Bonded parameters were assigned using the Sage small molecule force field, and electrostatics were treated using the AM1-BCC charge model^29^ generated using the OpenEye toolkits.^30^ Condensed phase training was run for ten ForceBalance iterations, and required 155 hours on 60 GPU workers.

Table 1 reports the root mean square errors (RMSE) of our optimised DE-based force field (named DE-FF) on the condensed phase training set, relative to experiment. DE-FF shows excellent performance across the training set, and shows a statistically significant performance improvement across all measures relative to the Sage LJ-based force field, which was trained on the same data (Section 4.2). In particular, the accuracy on the enthalpy of mixing set, which was used during training of Sage as a simple surrogate for how well the force field might do at predicting protein–ligand interactions, points to the future use of DE-FF in modelling complex interactions.

**Table 1:** Mixture training set accuracy for Sage and DE-FF, reported as the RMSE compared to experiment. Statistical measures are reported with 95% confidence intervals from 1000 iterations of bootstrapping with replacement

	Sage	DE-FF
Pure density (g mL^−1^)	0.030^0.036^_0.023_	0.023^0.028^_0.018_
Binary density (g mL^−1^)	0.014^0.016^_0.013_	0.012^0.013^_0.011_
Enthalpy of mixing (kcal mol^−1^)	0.128^0.143^_0.113_	0.097^0.109^_0.084_

The optimised DE-FF parameter set is available at https://github.com/jthorton/de-forcefields, and the changes in parameters relative to their initial values are shown in Fig. S2.† Starting from the trained values for the DE-B68 water model, the re-fit *α* and *β* parameters decrease from 16.789 to 16.766 and 4.529 to 4.427, respectively, which seems to justify their treatment as global fit parameters. Changes in *r*_*m*_ of >0.2 Å are observed in SMIRKS types ‘[#1 : 1]-[#7]’ and ‘[#35 : 1]’, which correspond to polar hydrogen bonded to nitrogen and bromine, respectively. All changes in *ε* are <0.01 kcal mol^−1^. Examples of changes in the non-bonded potentials, relative to Sage, are shown in Fig. S3.† While DE-FF is intended to be an effective force field for use in the condensed phase, it is useful to compare dimer potential energy curves against accurate quantum chemistry approaches in the gas phase. Exact agreement is not expected due to approximate treatment of electrostatics and polarisation by fixed charge force fields,^31^ but nevertheless DE-FF and Sage give root-mean-square errors of 1.65^1.93^_1.38_ and 1.48^1.70^_1.26_ kcal mol^−1^, relative to CCSD(T)/CBS for a subset of the DESS66x8 dataset covered by these force fields.^32^ Example comparisons of dimer dissociation curves are displayed in Fig. S4,† and the full set of potential energy surfaces is available in the ESI Data.†

We note here that co-optimisation of the small molecule and water force fields is not commonly performed, and some of the improvement in Table 1 may be due to this approach. For example, the Sage force field uses the standard TIP3P water model, with fixed parameters. A comparison of properties of water mixtures is given in Section S2† and confirms that the strong performance of DE-FF on the enthalpy of mixing training set is due in large part to improved descriptions of water – small molecule interactions. Fig. S5† further demonstrates that the overall accuracy of the water force field (tested on pure water properties) does not deteriorate significantly upon re-optimisation of the global *α* and *β* parameters, and is still suitable as a water model.

Following fitting of the DE-FF non-bonded parameters, the force field was completed by fitting the valence (bond, angle and torsion) parameters against curated quantum chemistry reference data. In this case, a subset of the Sage valence training dataset was employed by filtering out molecules for which DE-FF non-bonded parameters had not been optimised (Section 4.2). Valence training converged after six ForceBalance iterations, and required a total of 67 hours on 300 CPU workers. As expected, the changed treatment of the non-bonded interactions has little effect on the equilibrium bond lengths and force constants (Fig. S8 and S9†) and equilibrium angles (Fig. S10†). We do observe larger shifts of up to around 30% in the angle force constants (Fig. S11†), but the combined effect of these changes with the torsion parameters will be evaluated in the next section.

### 2.3 DE-FF is transferable to molecules and properties outside the training set

As an example measure of the ability of the valence parameters to extrapolate to molecules outside the training set, Table 2 reports the accuracy of the Sage and DE-FF force fields on a set of 671 torsion scans collected by fragmenting a set of 199 druglike molecules with diverse chemical moieties and a range of net charges.^34^ The same measures of force field accuracy are used as reported previously,^33^ namely a comparison between QM and MM optimised geometries and energetics. Perhaps surprisingly, given that we expect DE-FF to demonstrate more flexibility in the steepness of repulsive interactions as two atoms approach, the LJ- and DE-based methods perform identically, within statistical uncertainty. However, this is likely because the DE non-bonded parameters have not yet been trained on intramolecular energetics, and many details of the fit such as the initialised torsion parameters and treatment of 1–4 scaling are still “biased” towards LJ potentials. Further investigation of intramolecular energetics with the DE-FF and more flexibility in the fitting approach will likely be warranted. A full comparison between DE-FF and Sage conformational geometries and energetics for the standard Sage valence test set^23^ is shown in Fig. S12† and again reveals little difference between the two force fields.

**Table 2:** Performance of Sage and DE-FF Parameters on the fragmented Wang dataset.^33^ The “Max. RMSD” reports the maximum root mean square deviation between relaxed quantum chemistry and molecular mechanics geometries across a torsion scan (averaged over all scans). The “RMS Δ*E*” reports the root mean square difference between MM and QM potential energy surfaces

	Max. RMSD (Å)	RMS Δ*E* (kcal mol^−1^)
Sage (OpenFF 2.0.0)	0.65^0.70^_0.61_	1.10^1.14^_1.05_
DE-FF	0.65^0.69^_0.60_	1.16^1.22^_1.10_

**Fig. 3:**
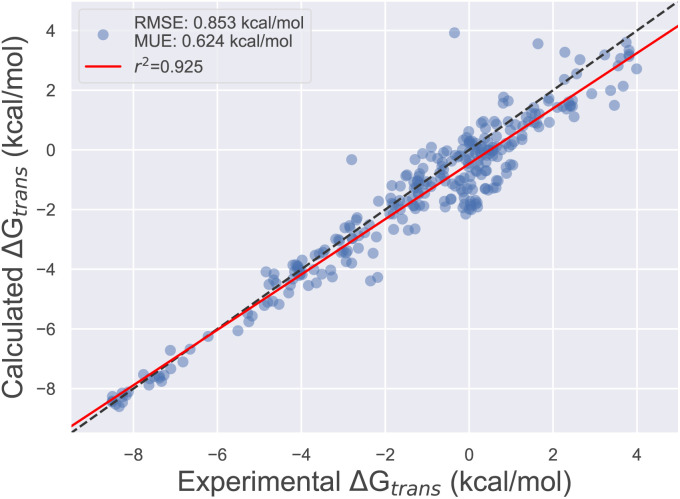
Comparison between transfer free energies computed using the DE-FF transferable small molecule force field, and experiment.

Using the final DE-FF valence and non-bonded parameter sets, we tested the accuracy on the Sage condensed phase benchmark set, which comprises 284 transfer free energies between aqueous and non-aqueous media (Section 4.3). This transfer of molecules between solvents of different polarities is expected to be a good surrogate for the accuracy of force fields for protein–ligand binding applications. Fig. 3 shows the correlation between experimental and computed transfer free energies for the DE-FF. The RMS error is just 0.85^0.97^_0.76_ kcal mol^−1^, which compares favourably with the Sage force field for the same dataset (RMSE 1.08^1.19^_0.99_ kcal mol^−1^). Full statistics and comparisons between the force fields are provided in Section S3.†

DE-FF performs well on enthalpy of mixing and transfer free energy benchmarks, but we did not train on properties that depend on vacuum energies (such as heats of vapourisation). We can extract the component aqueous and non-aqueous solvation free energies from the condensed phase benchmark set for DE-FF, and find RMS errors of 1.39^1.54^_1.25_ and 1.07^1.15^_0.99_ kcal mol^−1^, respectively. For comparison, GAFF and Sage have RMS errors in hydration free energy of 0.93^1.03^_0.83_ and 1.01^1.13^_0.88_ kcal mol^−1^, respectively (Section S3†). We note that we have used the same condensed phase dataset as used for training the Sage Lennard–Jones parameters, so it is interesting that Sage performs better for hydration free energy predictions. This may be because DE-FF inherits the fixed-charge, non-polarisable AM1-BCC charge model that has been co-developed with LJ force field models over the years. Co-training of electrostatic and DE parameters would be interesting to explore to further improve the accuracy of solvation free energies in future. However, in this first version, we are content to target transfer properties for use in condensed phase modelling.

### 2.4 Timing and convergence

Sampling performance was investigated using our implementation of the DE-FF in the OpenMM custom force class, which provides just-in-time compilation for energy and force calculations on CPUs or GPUs.^24^ For an ethanol molecule solvated in 1000 (4-point) water molecules, we obtain throughput of 185 ns per day with a Tesla V100 32G NVLink 2.0. Thus, run times are on the same order as similar calculations using the Sage force field with a soft core LJ potential for the ethanol molecule (255 ns per day), and significantly faster than a previously-reported CPU-only implementation in pmemd.^11^ We expect further optimisation to be possible, given that LJ interactions are computed using dedicated and optimised CUDA code in OpenMM. Similar optimisations could be performed for the DE functional form.

Section S4† further examines the natural soft-core properties of the DE-FF and the convergence of the reported free energy calculations with the *λ*-schedule. In brief, a naive linear scaling of the potential with *λ* causes convergence issues, but by additionally scaling down the *α* and *β* parameters of eqn (2) we obtain very similar convergence properties as the LJ soft-core potential (Section S4†). For both the LJ soft core and DE-FF, we are even able to pare the annihilation legs of the calculation down to six *λ* windows, from the default of 16, with <0.1 kcal mol^−1^ change in free energy (Table S7†). That is, the natural soft core of DE-FF shows very similar convergence properties to the optimised LJ soft core, without the additional coding complexity of changing functional form for free energy calculations.

## 3 Conclusions

Here, we have presented Smirnoff-plugins as a flexible framework to extend OpenFF style force fields with custom functional forms. Historically, training a new small molecule transferable force field for condensed phase simulations has been prohibitive due to the sheer number of fitting parameters that are required to cover chemical space. Interfacing Smirnoff-plugins with the OpenFF software stack allows us to assign force field parameters *via* direct chemical perception, which substantially reduces the number of redundant parameters to be re-fit. For example, the Sage library contains 167 rotatable torsion parameters, compared to the 146 K of OPLS3e.^33,35^ While this minimal parameter set does come with reduced accuracy compared to highly parameterised, proprietary force fields,^36^ users can make use of OpenFF-BespokeFit^33^ to fit bespoke torsion parameters to molecules of interest and improve agreement with QM data. The OpenFF software stack additionally includes robust tools, such as ForceBalance^19^ and Evaluator,^20^ which we have taken advantage of in the current study to automatically curate and train against thousands of experimental and quantum mechanical reference data points.

We have demonstrated the utility of Smirnoff-plugins by training new, transferable small molecule and water force fields using the recently-proposed double exponential non-bonded potential.^10,11^ The added flexibility of the DE-FF functional form was demonstrated through improved training metrics on pure liquid and binary densities, and enthalpies of mixing, when compared to the state-of-the-art OpenFF Sage LJ-based force field. Additional test set improvements in transfer free energies between aqueous and non-aqueous media were also observed (RMS errors of 0.85 and 1.08 kcal mol^−1^ for DE-FF and Sage, respectively), alongside comparable conformational energetics. This early success is particularly remarkable given that this is the first such general DE-FF, whereas LJ-based force fields are building on a long history of parameter optimisation and tuning. While currently modestly slower than the LJ soft-core potential, our GPU implementation of DE-FF in OpenMM offers substantial speed-up compared to previous CPU implementations.

There are many pathways to future improvement and applications of DE-FF. For a complete small molecule and biomolecular force field, training and testing of library torsion parameters will be required for proteins and other biomolecules. Again, this will be facilitated through interfaces with OpenFF infrastructure, and the first (LJ-based) OpenFF protein force field is planned for the next major (“Rosemary”) release. The accurate transfer free energies and natural soft-core of the DE-FF point to future use in protein–ligand binding free energy calculations for computer-aided design. Additional potential applications include the study of molecular phase diagrams, where behaviour of the potential over a wide pressure range is desirable.^37^ For completeness, DE-FF parameters will also be required for the SMIRKS types, including F, P, S, I and counter-ions, which are under-represented in the mixture training data at present,^23^ as well as those bonded parameters not currently covered by the Sage valence training dataset (Section 4.2). Additional quantum mechanical data for missing regions of chemical space will be relatively straightforward to collect and curate as part of future OpenFF updates. For condensed phase training and testing, as well as extending the number of mixture properties available in databases such as ThermoML, it should also be possible to add properties such as speed of sound, dielectric constants and crystallographic densities and sublimation enthalpies in future datasets.^20^ Training and testing against these new datasets can be performed using the documented procedures developed here.

Future improvements in fitting procedures should focus on co-optimisation of the electrostatic parameters (currently the AM1-BCC charge model is employed without change). We have used throughout this paper the Lorentz–Berthelot mixing rules to compute *ε* and *r*_*m*_ parameters for unlike atoms,^38,39^ but it would be interesting to test for alternative solutions in the context of the DE potential.^40^ Closer examination of the intramolecular, short-ranged properties of the DE potential may also lead to adjustments to, for example, the treatment of 1–4 interactions and improvements in conformational energetics. Here, we started the training procedure of DE-FF from initial *ε* and *r*_*m*_ parameters (eqn (2)) from the Sage LJ library, which may place limits on the achieved accuracy if parameter space is not adequately explored. In this regard, it may be beneficial to use more advanced, QM inspired functional forms as starting points,^8,41,42^ as we explored for the DE-B68 water model.^27^ More fundamentally, the automated training and testing procedures put forward here should facilitate much wider experiments in non-bonded functional form exploration. While we have used the previously suggested DE-FF as a proof-of-concept, it may be that more physically motivated functional forms, such as the B68 potential, are better able to model the balance between accurate behaviour in the bonding region and correct asymptotic behaviour at long range. To facilitate community exploration of new force field functional forms, we have made all software required to derive and use the designed force fields freely available at https://github.com/jthorton/de-forcefields.

## 4 Computational methods

### 4.1 Smirnoff-plugins

Smirnoff-plugins is an open source and highly flexible framework to extend SMIRNOFF-style force fields with custom interaction potentials. The energy expression is implemented in a non-bonded interaction class, and the package automatically handles scaled 1–4 non-bonded interactions *via* a custom bond force and has support for virtual sites. Currently Smirnoff-plugins offers implementations of the damped Buckingham-6-8 and double exponential potentials used in this work, but others may be trivially added. The package makes use of the openff-toolkits plugin framework which is able to use custom potential definitions to load parts of SMIRNOFF force fields that it does not recognise. In this case, the DE potential and its associated parameters would be loaded and applied to an OpenMM system by Smirnoff-plugins, with all other standard terms (electrostatic and valence) handled by the openff-toolkit. Thus, once the package is installed, users are able to use these custom force fields with very minor code changes to their current workflows. For example the following code could be used to load such a force field with the openff-toolkit:^43^
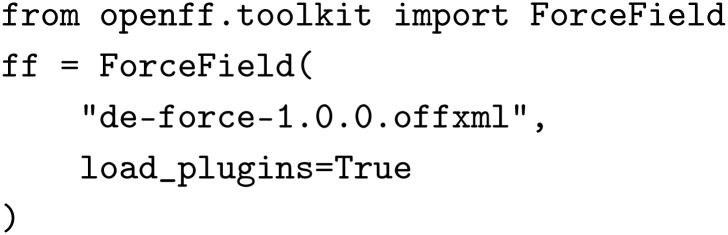
where the .offxml file contains all the valence and non-bonded parameters fit in the current study. Smirnoff-plugins then enables the use of custom functional form force fields with the entire OpenFF fitting software stack including Evaluator,^20^ ForceBalance^19^ and BespokeFit.^33^ Smirnoff-plugins is not limited to non-bonded interactions and can be used to supplement or replace any part of the force field. This will be critical for exploratory investigations into improving force field accuracy *via* new non-bonded and valence functional forms, such as Urey–Bradley cross coupling terms.

### 4.2 Training

#### 4.2.1 Water model

The DE water model was fit to reproduce six experimentally determined properties over a wide range of temperatures and pressures. The reference values of the properties: density, heat of vapourisation, thermal expansion coefficient, isothermal compressibility, isobaric heat capacity and dielectric constant are identical to those used in previous work fitting the TIP4P-FB and TIP3P-FB models.^26^ Due to computational constraints however we only included 12 data points in total distributed between temperatures of 249.15–373.15 K and the full pressure range, see the ESI Data† for the complete set of reference data used. The ForceBalance control file is provided in the ESI Data† but briefly the model was held rigid at the TIP4P-FB geometry *via* bond and angle constraints. The liquid simulations started with 1 ns of equilibration followed by a 8 ns production run using a 1 fs timestep. Gas phase simulations were equilibrated for 0.5 ns before a production simulation of 5 ns using a 0.5 fs time step.

#### 4.2.2 Non-bonded parameters

The basis of the physical property training dataset was sourced from the NIST ThermoML^44^ Archive *via* OpenFF-Evaluator and is identical to that used in the training of OpenFF-Sage,^23^ except for the addition of six pure water densities distributed between temperatures of 281.15–359.15 K at standard pressure. These properties were included to regularise the water parameters to ensure the pure water properties were not degraded during training. The final training dataset consisted of 76 neat liquid densities, 485 densities of binary mixtures and 477 enthalpies of mixing of binary mixtures. The complete training dataset can be found in the ESI Data† in a format readable by Evaluator.^20^

All bonds involving hydrogen were constrained during the simulations. Polar hydrogens had their vdW contribution removed by setting *ε* = 0 as the very small parameters caused slow convergence of the long-range correction in OpenMM-7.6.0 which caused the simulations to fail. This was deemed to have a negligible impact on the simulation accuracy as the parameters are placeholders designed to be “small but non-zero” in OpenFF-Sage^23^ from which they were transferred. The parameters of DE-FF were then optimised using ForceBalance,^19^ which minimises a weighted least squares objective function comparing the calculated and experimental condensed phase properties. The construction of the objective function was exactly the same as that used in the training of OpenFF-Sage^23^ and the ForceBalance input files can be found in the ESI Data.†

#### 4.2.3 Bonded parameters

Valence parameters (bonds, angles and proper torsions) were optimised after the non-bonded optimisation procedure described above. The QM target reference dataset comprised a subset of the total data used to fit OpenFF-Sage and was extracted from QCArchive^45^ using QCSubmit.^33^ Any molecules which would be parameterised with unoptimised non-bonded parameters (S, P, F, I) were filtered out of the dataset. Starting from the OpenFF-Sage valence parameters, the harmonic bond equilibrium length and force constant, harmonic angle equilibrium angle and force constant and proper torsion force constants were optimised using ForceBalance.^19^ This resulted in the following numbers of parameters to be optimised:

• Harmonic bond stretches: 42 out of 88 parameters were optimised.

• Harmonic angle bending: 25 out of 40 parameters were optimised.

• Proper torsions: 53 out of 167 torsion parameters were optimised.

Improper torsions were retained at their Sage values along with the 1–4 non-bonded scaling factor. ForceBalance was used to then optimise the parameters to reduce structural and energetic differences between the accurate QM reference data and the MM predicted values. The fitting used exactly the same optimisation settings as the fitting of OpenFF-Sage. Input and output files may be found in the ESI Data.† To enable a fair comparison of the final DE-FF with a LJ-based FF, we also trained an alternative Sage-style force field to the same reduced target dataset. All non-bonded parameters were held fixed and the same valence terms were optimised starting from their Sage values. The resulting force field performed similarly to Sage on a geometry and conformational energetics test set (Fig. S12†), and so we employ the standard Sage (OpenFF 2.0.0) in the remaining tests reported in the main text.

### 4.3 Testing

#### 4.3.1 Non-bonded parameters

The Absolv package^46^ was developed as part of this project and offers a simple API for computing the change in free energy upon transferring a solute from one solvent to another, or to vacuum in the case of solvation free energy calculations. It offers two routes to this end: standard equilibrium, and non-equilibrium switching, calculations. Most importantly, by using OpenMM as the MD engine, the framework allows for free energies to be computed using arbitrary functional forms implemented *via* the OpenMM custom non-bonded class.^24^ Users are free to define their force field functional form and any special treatment to be applied during the calculation such as the soft-core approach to the LJ potential as part of the calculation setup. Absolv is compatible with the OpenFF software stack making it straightforward to test new functional forms implemented *via* Smirnoff-plugins. Example scripts used to set up the calculations are included in the ESI Data.† To ensure the correctness of the default protocols offered by Absolv, regression tests were performed using systems previously studied to determine the reproducibility of free energy calculations between different software.^47^ For the nine free energy calculations run in triplicate we find the results from Absolv to be within error bars of the values reported by other packages (Fig. S20†).

The benchmark solvation free energy datasets were constructed from subsets of those used in the testing of OpenFF-Sage.^23^ The non-aqueous solvation free energy dataset extracted from the MNSol database^48^ was first filtered to only include solute–solvent pairs which would be parameterised with the 15 SMIRKS exercised during the fitting of DE-FF. Then we filtered out all solutes that did not have an aqueous solvation free energy in the FreeSolv database.^49^ This resulted in 72 unique solutes and 284 unique solute–solvent pairs from which we computed the aqueous solvation free energies (Δ*G*_solv_(aq)), and non-aqueous solvation free energies (Δ*G*_solv_(nonaq)), respectively. We then computed 284 aqueous to non-aqueous transfer free energies from the individual components *via*:

4 Δ*G*_trans_(aq → nonaq) = Δ*G*_solv_(nonaq) − Δ*G*_solv_(aq)

The free energy calculations were set up using Absolv-0.0.1 and used a 20-window *λ*-schedule to first remove the electrostatics followed by the vdW solute–solvent interactions for a simulation box containing 1000 solvent molecules and a single solute. In total, 2000 equilibrium data points were collected from each *λ*-window during the production simulations, and free energies were estimated using MBAR.^50^ The AM1-BCC charging scheme, consistent with mainline OpenFF force fields (Parsley, Sage) was used *via* the OpenEye toolkit (2022.1.1).^30^ The Lorentz–Berthelot combination rules were used to compute the interaction parameters between two, unlike atoms. Constraints were placed on all bonds involving hydrogen and a long-range energy correction was used.^51^ Further computational details and convergence tests are available in ESI Section S5.†

#### 4.3.2 Bonded parameters

The valence benchmark dataset used in this work was made from a subset of the recently published OpenFF Industry Benchmark Season 1 dataset^36^ on QCArchive.^45^ Any molecules which would be parameterised with unoptimised non-bonded parameters (S, P, F, I) were filtered out of the dataset. This resulted in a final dataset containing 5062 unique molecules and 37 259 conformers spanning a range of formal charges and molecular weights. The dataset covers a wide range of chemistry and therefore exercises many more parameters than were optimised during the valence fitting performed here. A breakdown of the parameter coverage for each valence type is listed below:

• Harmonic bond stretches: 45 out of 88 total parameters used.

• Harmonic angle bending: 26 out of 40 total parameters used.

• Proper torsions: 118 out of 167 total parameters used.

For parameters not met in the training set, we used the default parameters from the Sage parameter library, hence further improvement in conformational energetics is likely possible through expansion of the training set. The benchmarking was performed using the workflow first described by Lim *et al.*^52^ and the scripts and inputs required to repeat it can be found in the ESI Data.†

## Data availability

ESI Data,† including scripts used to curate the training and test data sets, input files required to reproduce the force field training and benchmarking, and scripts used to generate the input schemas and files and to perform ancillary data analysis, are available at https://github.com/jthorton/double-exp-vdw.

## Author contributions

Joshua Horton: conceptualisation, data curation, investigation, methodology, software, validation, visualisation, writing – review & editing. Simon Boothroyd: conceptualisation, data curation, investigation, methodology, software, validation, visualisation. Pavan Kumar Behara: investigation, validation, visualization, writing – review & editing. David Mobley: conceptualisation, funding acquisition, methodology, project administration, resources, supervision, writing – review & editing. Daniel Cole: conceptualisation, funding acquisition, methodology, project administration, resources, supervision, writing – original draft, writing – review & editing.

## Conflicts of interest

D. L. M serves on the scientific advisory boards of OpenEye Scientific Software and Anagenex, and is an Open Science Fellow with Psivant Sciences.

## Supplementary Material

DD-002-D3DD00070B-s001
