# Supplementary material for: A transferable double exponential potential for condensed phase simulations of small molecules
Source: Digit Discov. 2023 Jul 10;2(4):1178–87. doi: 10.1039/d3dd00070b (PMC10408570; doi:10.1039/d3dd00070b)
Supplement: DD-002-D3DD00070B-s001 [file DD-002-D3DD00070B-s001.pdf]

Electronic Supplementary Material (ESI) for Digital Discovery.
This journal is © The Royal Society of Chemistry 2023

Supporting Information for: A transferable double exponential potential for condensed phase simulations of small molecules

Joshua T. Horton,[†] Simon Boothroyd,[‡] Pavan Kumar Behara,[¶] David L. Mobley,[¶,§] and Daniel J. Cole[*,†]

†*School of Natural and Environmental Sciences, Newcastle University, Newcastle upon Tyne NE1 7RU, United Kingdom*

‡*Boothroyd Scientific Consulting Ltd., London WC2H 9JQ, United Kingdom*

¶*Department of Pharmaceutical Sciences, University of California, Irvine, California 92697, United States*

§*Department of Chemistry, University of California, Irvine, California 92697, United States*

E-mail: daniel.cole@ncl.ac.uk

S1 The Buckingham 6-8 Potential

The water model used as a starting point for our DE fit in this work is a four-point model, employing the Buckingham 6-8 non-bonded potential:[1]

$$U^{B68}(r) = A\exp(-br) - f_{damp,6}(r)\frac{C_6}{r^6} - f_{damp,8}(r)\frac{C_8}{r^8} \quad (1)$$

Here, Pauli repulsion is modelled by the first exponential term, and the leading order terms of the attractive dispersion interaction (C_6 and C_8) are retained.[2] The basic form of the Buckingham 6-8 potential diverges at $r = 0$, and so the dispersion interactions are damped using an incomplete gamma function:[3]

$$f_{damp,n}(r) = 1 - \exp(-\zeta r)\sum_{k=0}^{n}\frac{(\zeta r)^k}{k!} \quad (2)$$

with $\zeta = 35.8967$ nm^{-1} for the B68 water model.[1]

Figure S1 shows the O–O interaction potential for the B68 water model, using previously published A, b, C_6 and C_8 force field parameters.[1] Also plotted is a DE potential with parameters obtained from a non-linear least-squares fitting to the B68 potential. The resulting DE-B68 parameters (fit) are tabulated in Table S1. As expected from the overall shapes of the potential energy surfaces (Figure 1 in main text), the DE fit parameters are similar to the previously used DE-TIP3P parameters.[4] In particular, the DE-B68 potential has a nearly identical O–O equilibrium separation distance (r_m) and a larger well depth (ϵ), as compared to DE-TIP3P.

Table S1 also shows the same parameters after training to water condensed phase properties (DE-B68 (trained)). Little change from the initial fit parameters are required to recover the condensed phase data, as expected since the underlying B68 model has been trained against the same data. Also noteworthy is the similarity between the final, trained electrostatic parameters (O–X and q_H) and those of the LJ-based TIP4P-FB water model.

We have also tested the performance of our implementation of the DE-B68 water model, accessed through the smirnoff-plugins interface to OpenMM.[5] For a box of 1000 4-point water molecules, we obtain throughput of 225 ns/day with a Tesla V100 32G NVLink 2.0. This compares favourably with a throughput of 130 ns/day for the more complex B68 model on the same resources. Thus, the flexibility of the DE functional form has the potential to combine good accuracy (see Figure 2 in main text) with acceptable computational cost.

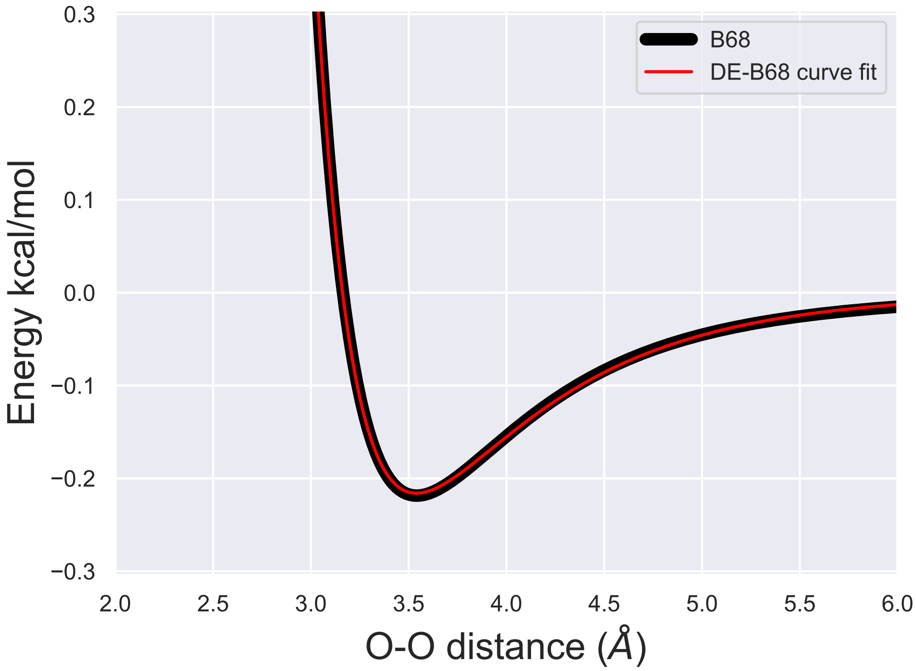

Figure S1: Comparison of O–O non-bonded interactions in water. The non-bonded component of the interaction energy (excluding electrostatics) is shown for the B68[1] and DE curve fit models. The B68 model is shown as a thicker line to help with visibility as the potentials become indistinguishable around the minimum of the potential.

Table S1: Force field parameters of four water models. Parameters are as defined in eqs 1 and 2 in the main text. O–X is the distance between oxygen and the off-site charge in 4-point water models, and q_H is the charge on the H atom.

	DE-TIP3P[4]	TIP4P-FB[6]	DE-B68 (fit)	DE-B68 (trained)
α	18.7	–	16.83	16.789
β	3.3	–	4.52	4.529
ϵ / kcal/mol	0.152	0.179082	0.216	0.21104
r_m / Å	3.5366	3.553	3.54	3.5204
O–X / Å	–	0.10527	–	0.10743
q_H / e	0.417	0.52587	–	0.53254

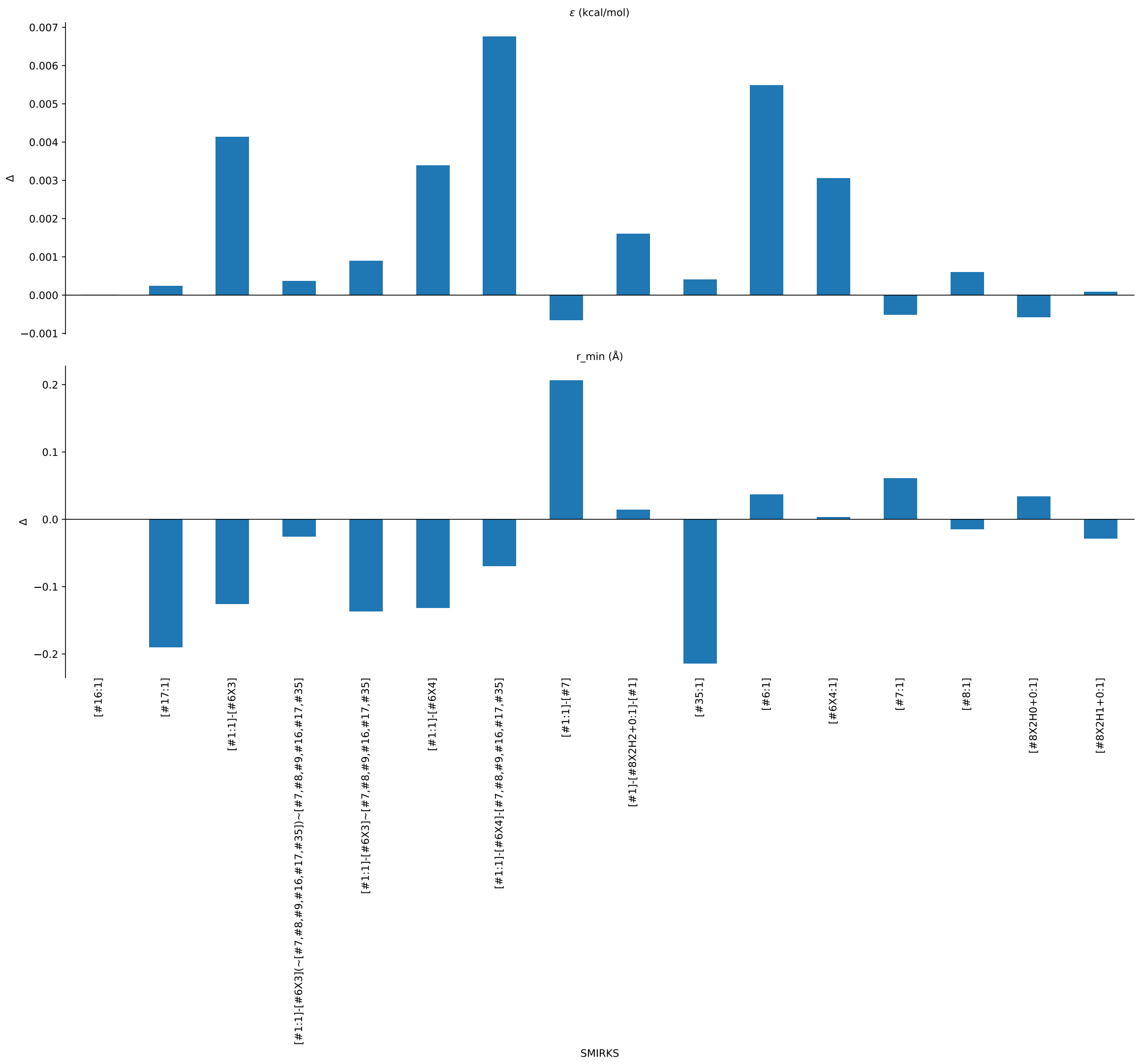

Figure S2: Change in force field parameters during the fitting of the DE model. The ϵ and r_m parameters for 15 OpenFF SMIRKS types, plus the parameters for water were allowed to vary.

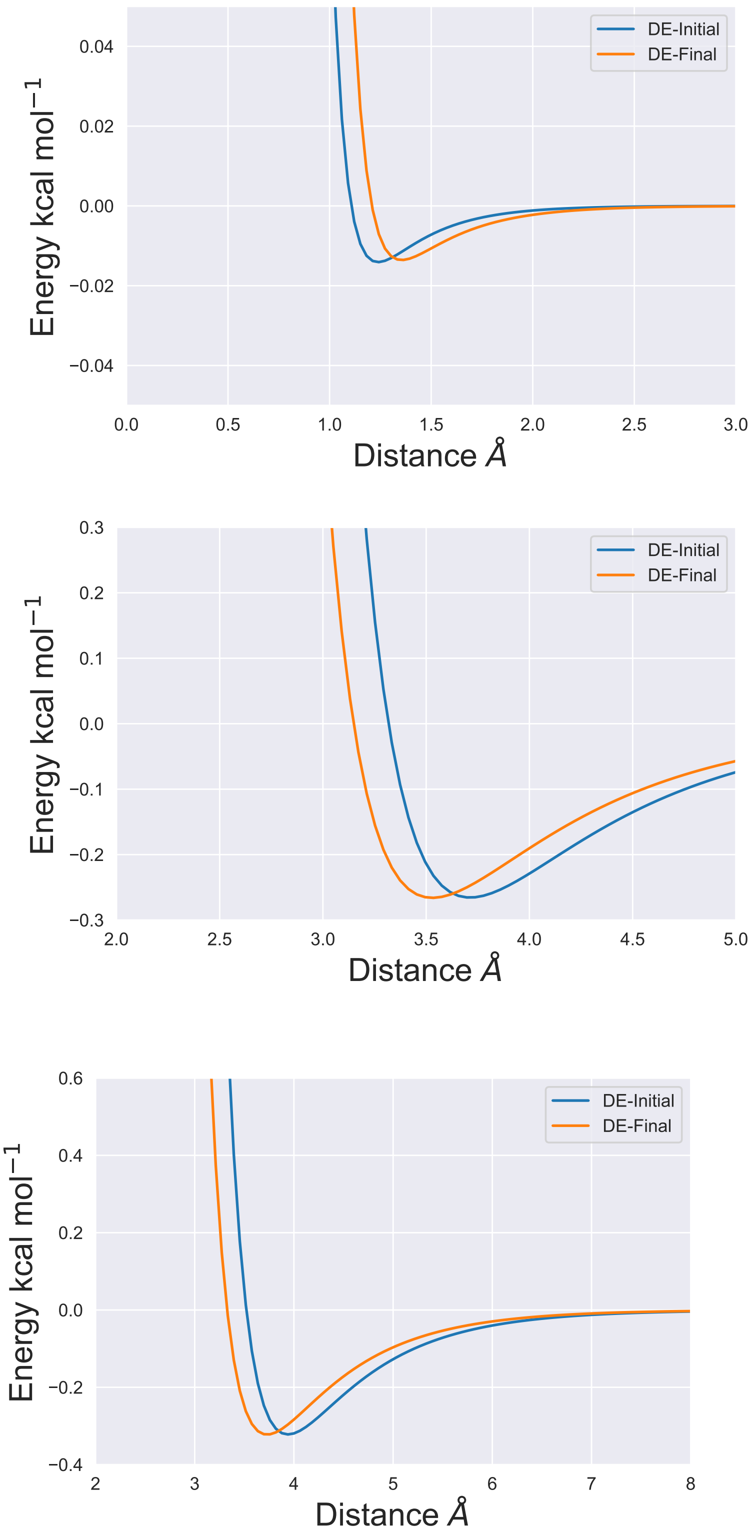

Figure S3: The change in the non-bonded potentials for the DE-FF as a result of fitting to physical properties is shown for SMIRKS types '[#1:1]-[#7]' (top), '[#17:1]' (middle) and '[#35:1]' (bottom).

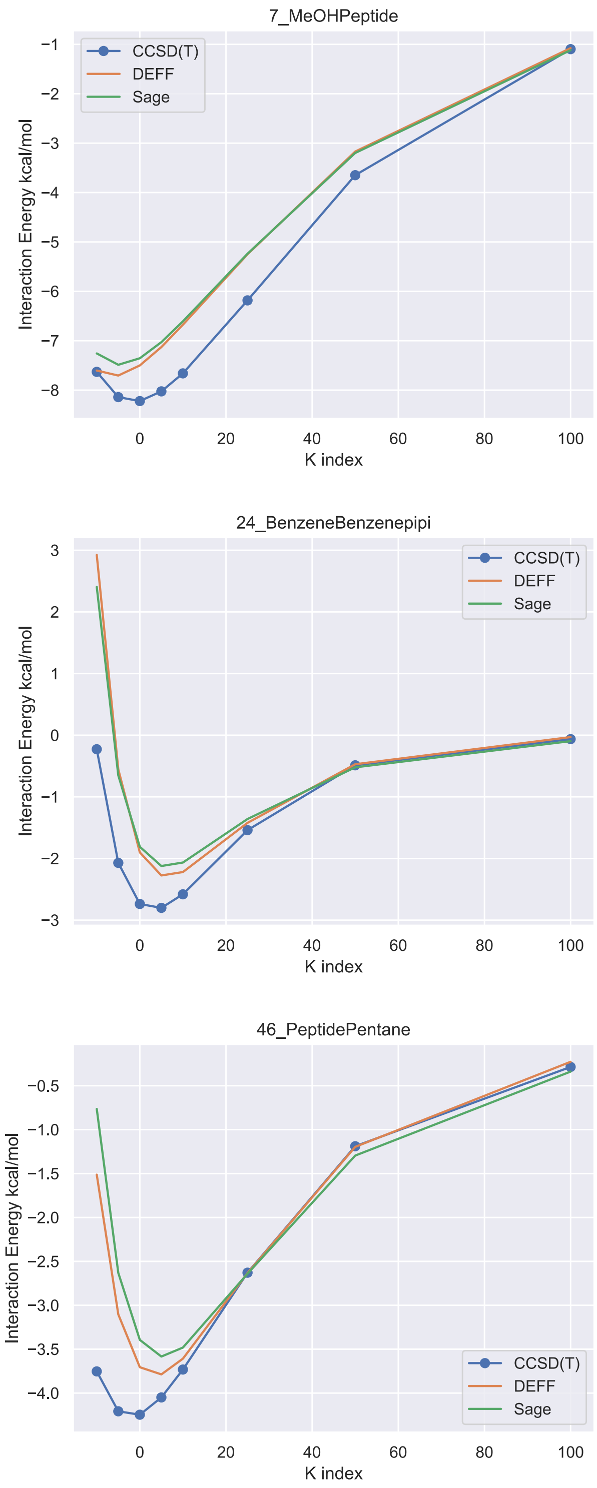

Figure S4: Comparisons between DE-FF, Sage and quantum chemistry (CCSD(T)/CBS) gas-phase dimer dissociation curves. Examples from the DESS66x8 dataset[7] are shown for polar-polar (methanol and N-methylacetamide, top), non-polar-non-polar (benzene dimer, middle), and polar-non-polar (N-methylacetamide and pentane, bottom). The full set of 59 plots is provided in the Supporting Data.

S2 Analysis of the co-optimised DE-B68 water model

In the training of the DE-FF non-bonded parameters, we chose to co-optimise the parameters of the DE-B68 water model (starting from the trained parameters in Table S1). It is possible that the parameters will significantly change such that it is no longer suitable as a pure liquid water model. To guard against this possibility, we also included pure water density target data in the fitting of DE-FF. Figure S5 confirms that the final water model is still sufficiently accurate. The biggest change is in the density (probably because we did not change the electrostatics of the water model), but even this changes by less than 1 % at room temperature.

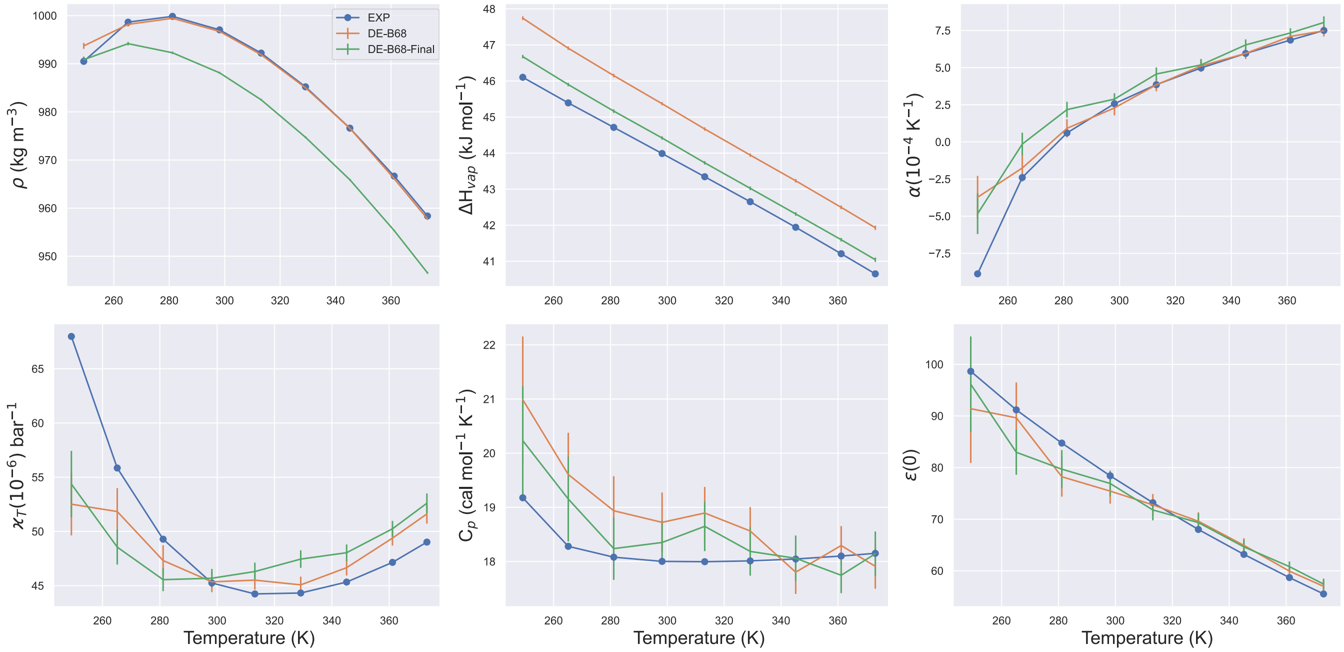

Figure S5: Water physical properties at a range of temperatures. Series of ForceBalance single point property calculations for the DE-B68 and DE-B68-Final (after mixture property fitting) models. All properties were used in the fitting of the DE-B68 model, with only pure density at temperatures of 281.15K, 298.15K, 313.15K, 329.15K, 343.15K and 359.15K included in the DE-FF fitting to regularise the water parameters.

From the overall statistics presented in Table 1 in the main text, we also separated out the enthalpy of mixing data for mixtures involving water (Table S2 and Figure S6), and those not involving water (Table S3 and Figure S7). These confirm that much of the improvement in this quantity comes from the improved description of interactions between small molecules and water, described by the DE-FF and co-optimised DE-B68 models respectively.

Table S2: Enthalpy of mixing for mixtures from the training set involving water (n=57), with 95% confidence intervals from 1000 iterations of bootstrapping with replacement.

Force Field	MUE (kcal/mol)	RMSE (kcal/mol)	r^2	τ	MSE (kcal/mol)
DE-FF	$0.104_{0.0811}^{0.127}$	$0.137_{0.101}^{0.174}$	$0.885_{0.803}^{0.940}$	$0.765_{0.675}^{0.844}$	$0.0664_{0.0349}^{0.0992}$
Sage[8]	$0.239_{0.205}^{0.273}$	$0.275_{0.240}^{0.309}$	$0.715_{0.512}^{0.836}$	$0.545_{0.358}^{0.684}$	$0.202_{0.154}^{0.249}$

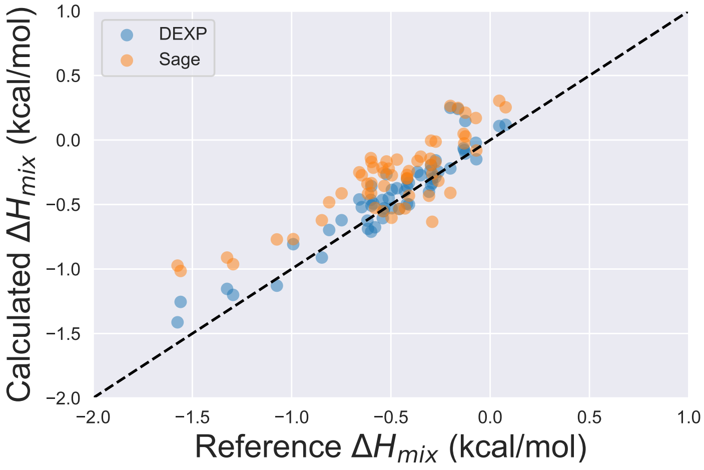

Figure S6: Enthalpy of mixing performance for mixtures in the training set involving water (n=57).

Table S3: Enthalpy of mixing for mixtures from the training set not involving water (n=420), with 95% confidence intervals from 1000 iterations of bootstrapping with replacement.

Force Field	MUE (kcal/mol)	RMSE (kcal/mol)	r^2	τ	MSE (kcal/mol)
DE-FF	$0.0590^{0.0656}_{0.0526}$	$0.0896^{0.104}_{0.0755}$	$0.725^{0.795}_{0.653}$	$0.724^{0.754}_{0.694}$	$0.0238^{0.0324}_{0.0156}$
Sage[8]	$0.0578^{0.0651}_{0.0517}$	$0.0909^{0105}_{0.0767}$	$0.719^{0.790}_{0.641}$	$0.727^{0.758}_{0.693}$	$0.0158^{0.0241}_{0.00737}$

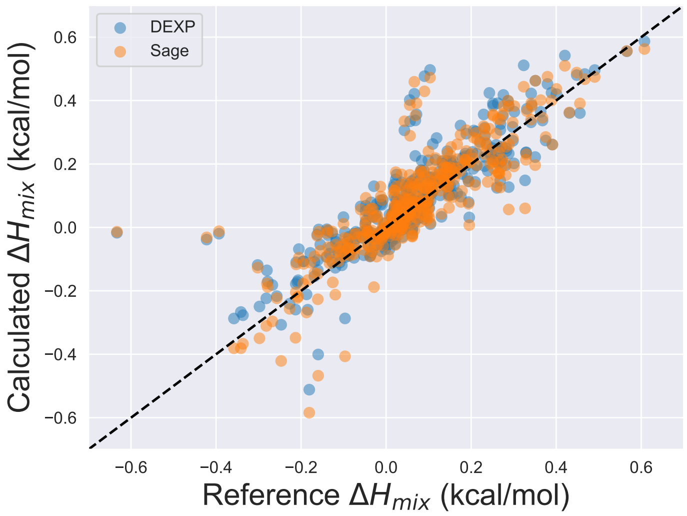

Figure S7: Enthalpy of mixing performance for mixtures in the training set not involving water (n=420).

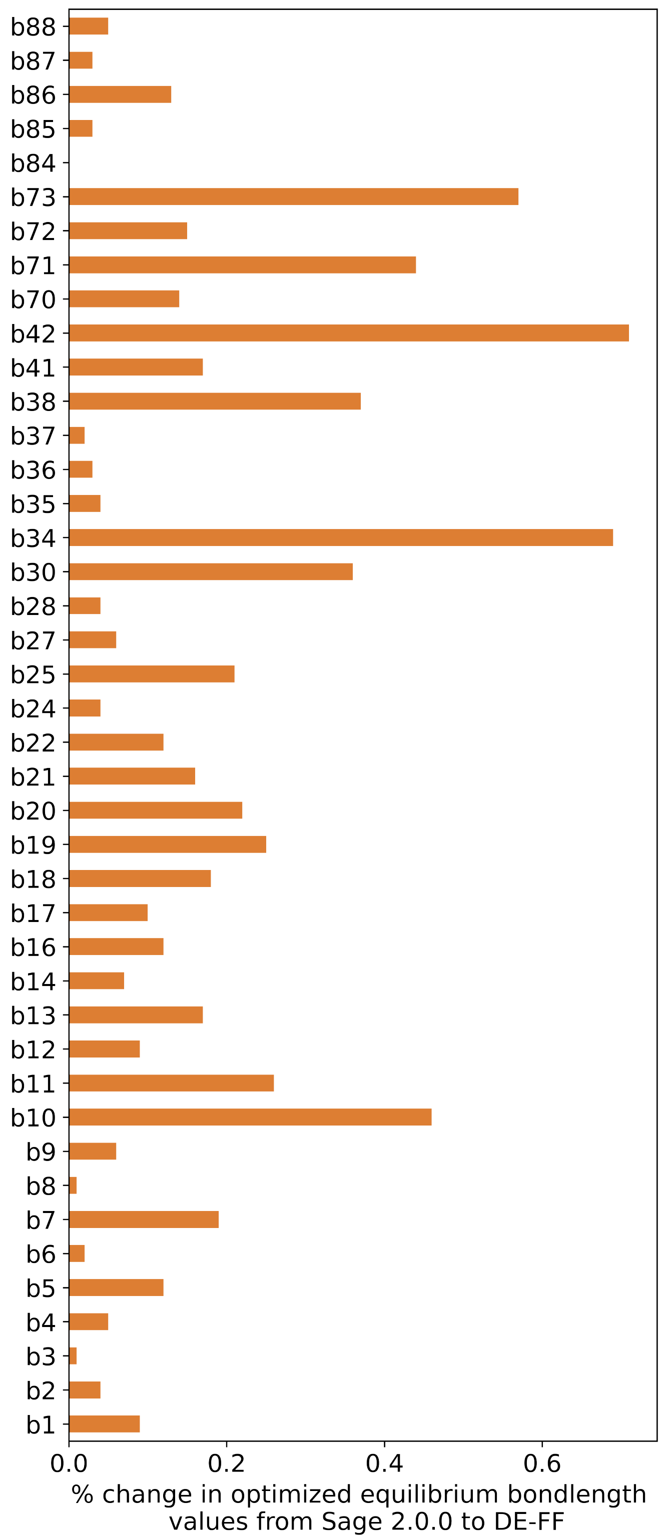

Figure S8: Change in trained equilibrium bond lengths between the Sage 2.0.0 and DE-FF small molecule force fields.

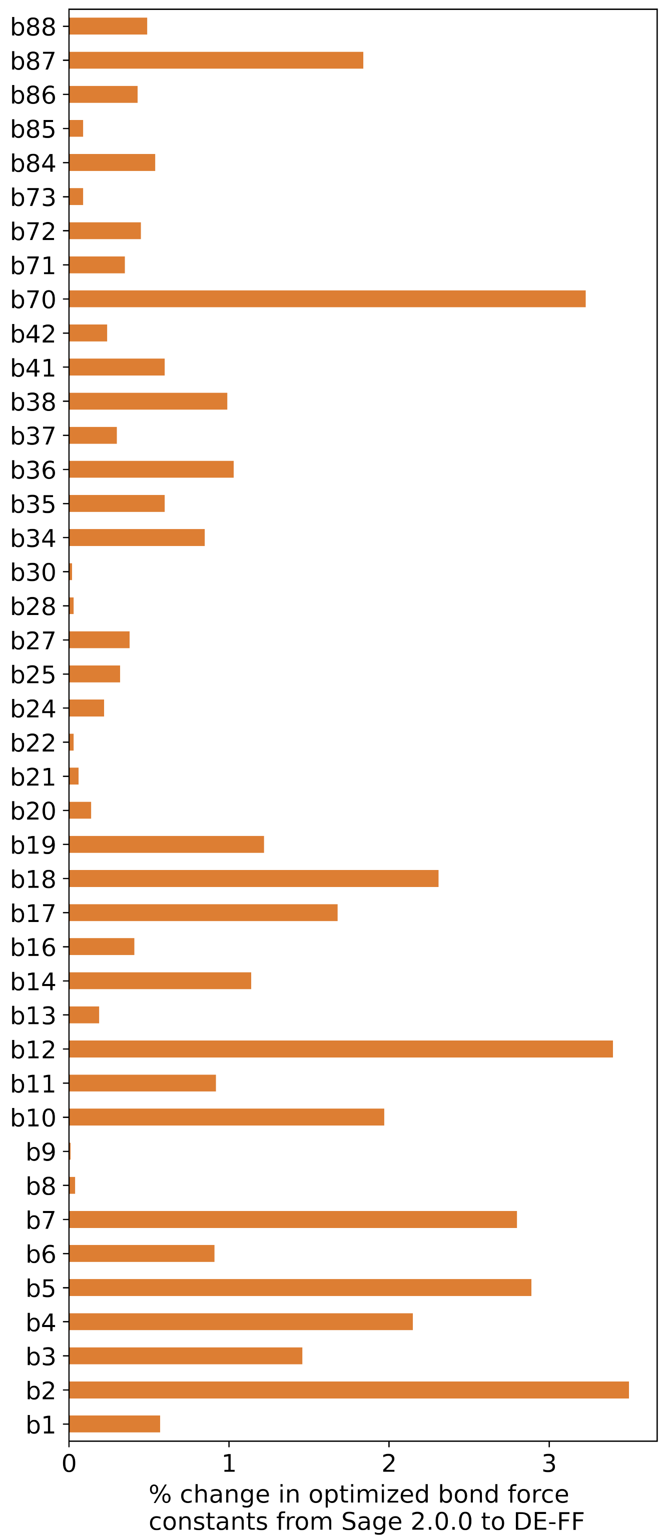

Figure S9: Change in trained bond force constants between the Sage 2.0.0 and DE-FF small molecule force fields.

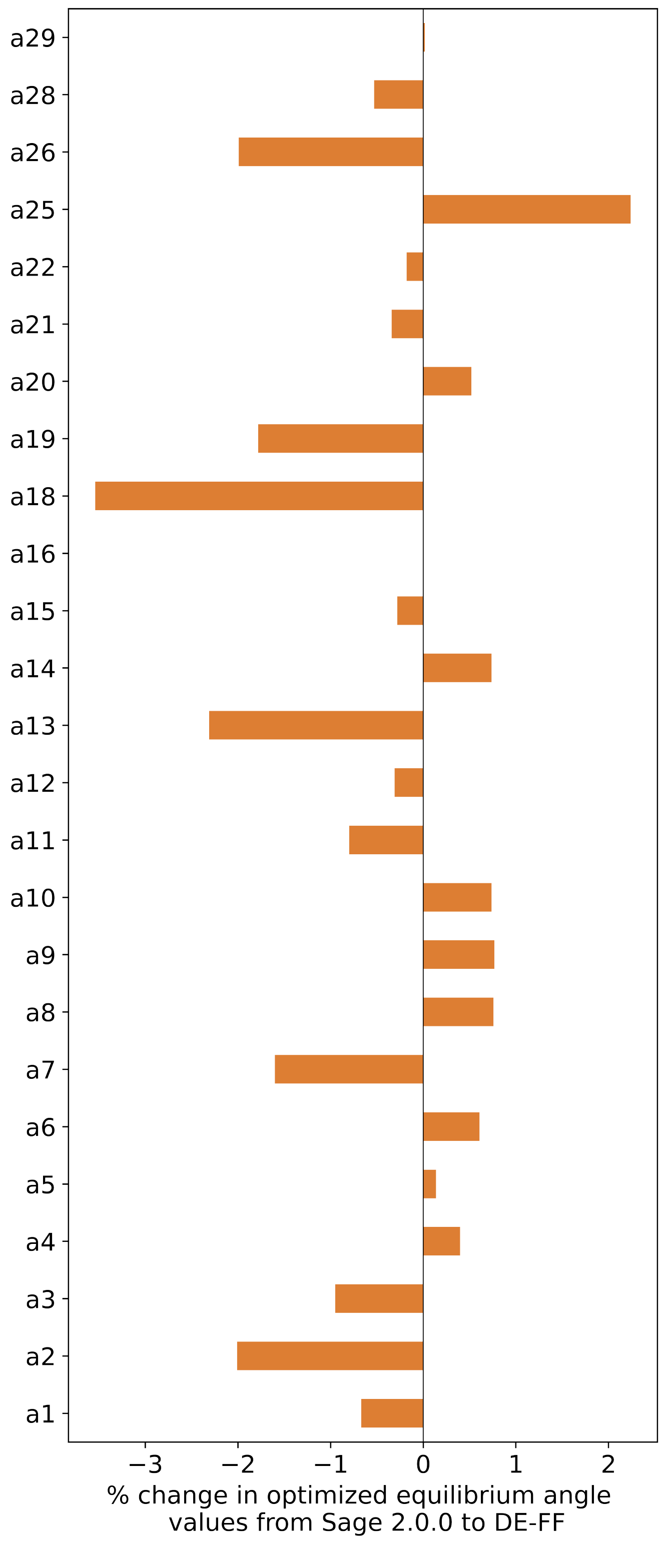

Figure S10: Change in trained equilibrium bond angles between the Sage 2.0.0 and DE-FF small molecule force fields.

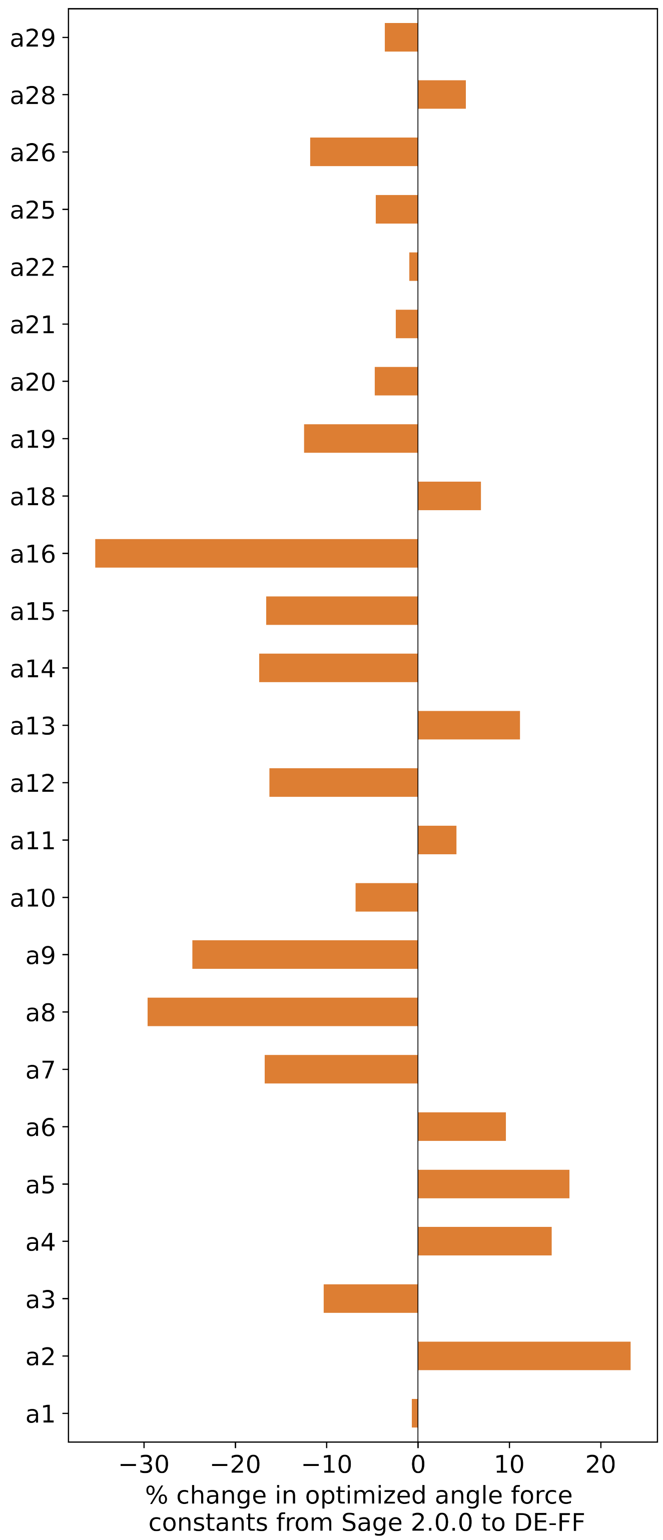

Figure S11: Change in trained angle force constants between the Sage 2.0.0 and DE-FF small molecule force fields.

S3 Test Set Accuracy

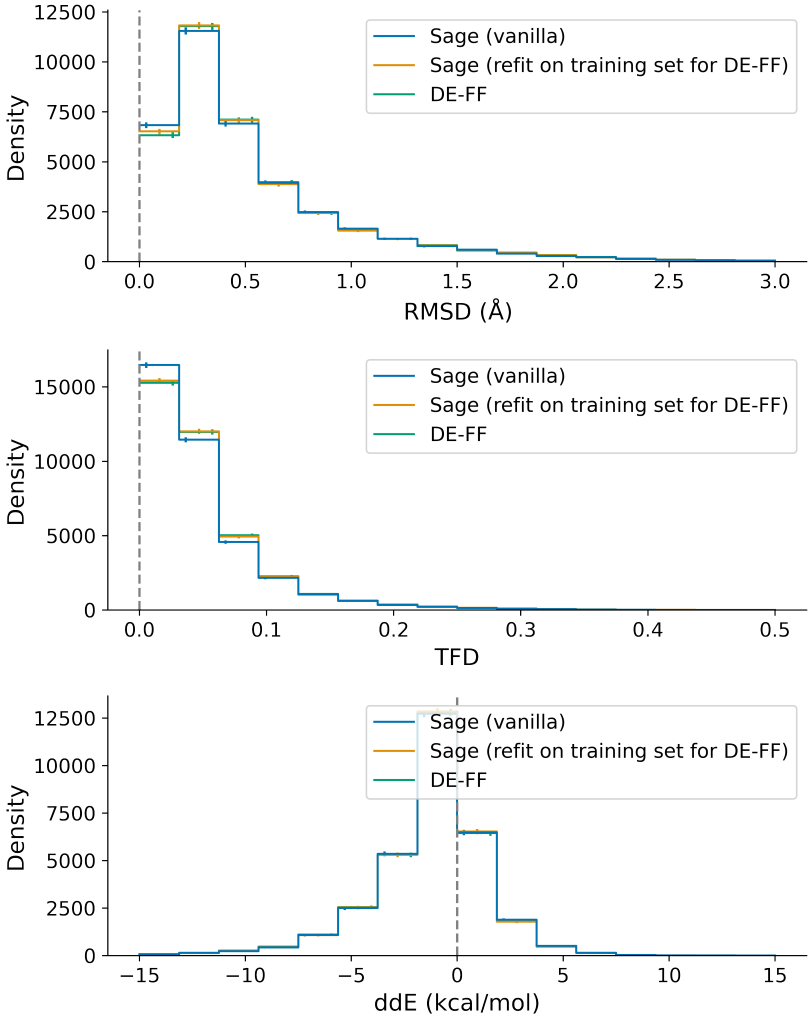

Figure S12: Step plots comparing geometries and conformational energetics of Sage 2.0.0 and DE-FF small molecule force fields with quantum mechanics. The error bars are bootstrapped errors for each bin. (Top) Root mean square deviation (RMSD) in geometries between MM optimised and QM optimised conformers. (Centre) Torsion fingerprint deviation (TFD), which is a weighted metric of deviations in dihedral angles.[8] (Bottom) Errors in relative conformer energies, with respect to the QM minimum energy conformer, for each molecule (ddE).[8]

Table S4: Hydration free energies computed using three different force fields (n=72), with 95% confidence intervals from 1000 iterations of bootstrapping with replacement.

Force Field	MUE (kcal/mol)	RMSE (kcal/mol)	r^2	τ	MSE (kcal/mol)
DE-FF	$1.259^{1.397}_{1.126}$	$1.39^{1.537}_{1.246}$	$0.930^{0.964}_{0.886}$	$0.791^{0.856}_{0.718}$	$1.19^{1.354}_{1.038}$
Sage[8]	$0.854^{0.979}_{0.730}$	$1.014^{1.131}_{0.878}$	$0.889^{0.931}_{0.823}$	$0.708^{0.797}_{0.607}$	$0.431^{0.636}_{0.210}$
GAFF[9]	$0.790^{0.904}_{0.683}$	$0.931^{1.033}_{0.830}$	$0.905^{0.941}_{0.839}$	$0.686^{0.791}_{0.559}$	$0.438^{0.627}_{0.245}$

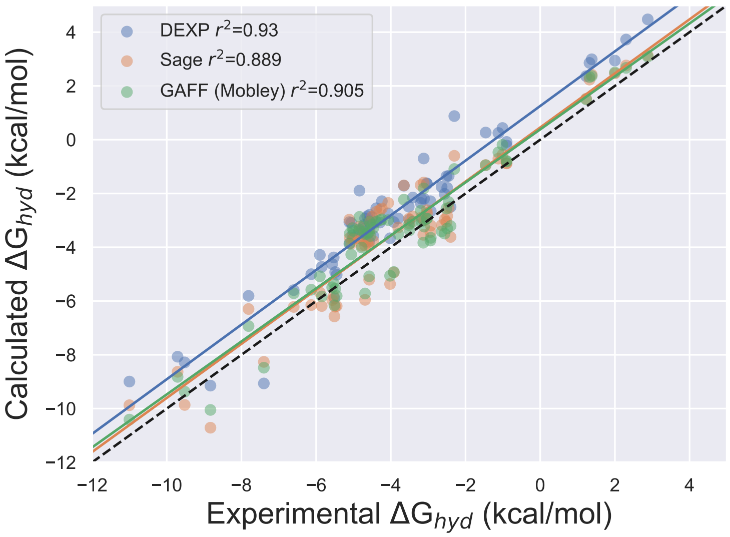

Figure S13: Hydration free energies (n=72) for Sage,[8] DE-FF and AM1-BCC-GAFF.[9]

Table S5: Non-aqueous solvation free energies (n=284), with 95% confidence intervals from 1000 iterations of bootstrapping with replacement.

Force Field	MUE (kcal/mol)	RMSE (kcal/mol)	r^2	τ	MSE (kcal/mol)
DE-FF	$0.900_{0.839}^{0.967}$	$1.066_{0.992}^{1.146}$	$0.869_{0.826}^{0.905}$	$0.739_{0.699}^{0.775}$	$0.866_{0.797}^{0.937}$
Sage[8]	$0.499_{0.450}^{0.548}$	$0.656_{0.582}^{0.728}$	$0.871_{0.823}^{0.902}$	$0.756_{0.720}^{0.789}$	$-0.008_{-0.080}^{0.069}$

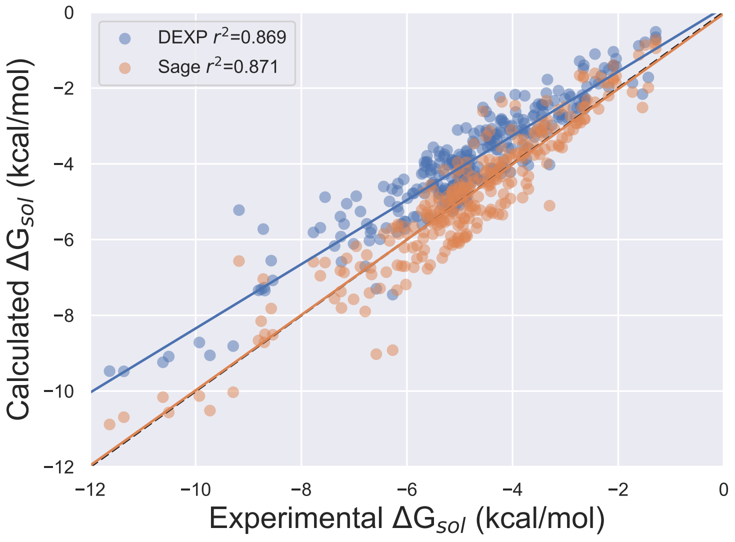

Figure S14: Non-aqueous solvation free energy calculations (n=284) computed using Sage and DE-FF.

Table S6: Non-aqueous to aqueous transfer free energies (n=284), with 95% confidence intervals from 1000 iterations of bootstrapping with replacement.

Force Field	MUE (kcal/mol)	RMSE (kcal/mol)	r^2	τ	MSE (kcal/mol)
DE-FF	$0.624_{0.560}^{0.696}$	$0.853_{0.755}^{0.965}$	$0.925_{0.890}^{0.948}$	$0.778_{0.733}^{0.818}$	$-0.377_{-0.285}^{-0.460}$
Sage[8]	$0.833_{0.753}^{0.920}$	$1.084_{0.986}^{1.189}$	$0.891_{0.848}^{0.922}$	$0.733_{0.682}^{0.782}$	$-0.566_{-0.455}^{-0.672}$

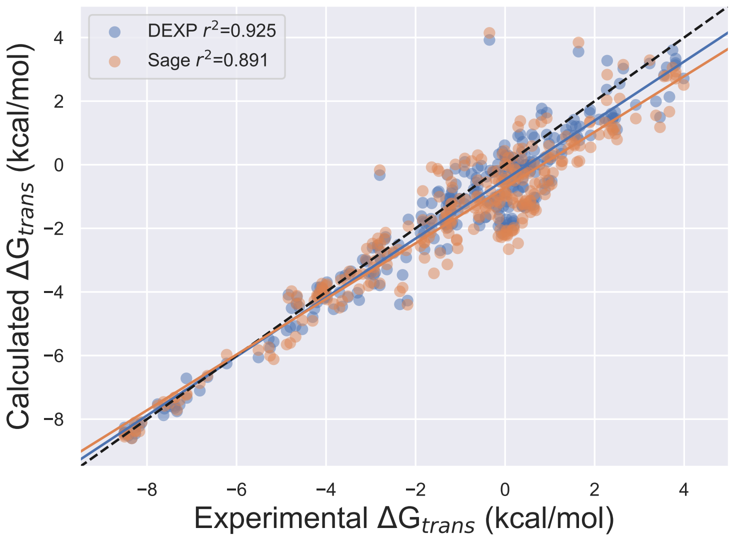

Figure S15: Non-aqueous to aqueous transfer free energies (n=284) computed using Sage and DE-FF.

S4 Convergence of Free Energy Calculations

The widely-used LJ soft-core potential is given by:[10]

$$V(r_{ij}) = \lambda \epsilon_{ij} \left[\left(\frac{r_{m,ij}}{r_{sc,ij}} \right)^{12} - 2 \left(\frac{r_{m,ij}}{r_{sc,ij}} \right)^{6} \right], \tag{3}$$

where $r_{sc,ij} = r_{m,ij} \left(0.25(1-\lambda) + (r_{ij}/r_{m,ij})^6\right)^{\frac{1}{6}}$. Figure S16(a) shows a typical variation of the potential energy surface with λ, including a gradual decrease to zero as the potential is turned off. Since the DE-FF has no singularity at $r = 0$, we might expect to be able to linearly interpolate the potential to zero:

$$V(r_{ij}) = \lambda \epsilon_{ij} \left[\frac{\beta e^{\alpha}}{\alpha - \beta} exp\left(-\alpha \frac{r_{ij}}{r_{m,ij}}\right) - \frac{\alpha e^{\beta}}{\alpha - \beta} exp\left(-\beta \frac{r_{ij}}{r_{m,ij}}\right) \right] \tag{4}$$

However, scaling the DE-FF potential in this way leads to a sudden reduction in the potential at small values of λ (Figure S16(b)). This would manifest as poor convergence in free energy calculations, due to poor phase space overlap at the λ end states (Figure S17).

Instead, we choose a scaling of the DE-FF that more closely resembles the behaviour of the LJ soft-core potential:

$$V(r_{ij}) = \lambda \epsilon_{ij} \left[\frac{\beta_s e^{\alpha_s}}{\alpha_s - \beta_s} exp\left(-\alpha_s \frac{r_{ij}}{r_{m,ij}}\right) - \frac{\alpha_s e^{\beta_s}}{\alpha_s - \beta_s} exp\left(-\beta_s \frac{r_{ij}}{r_{m,ij}}\right) \right], \tag{5}$$

where $\alpha_s = (1.1 + \lambda(\alpha - 1.1))$ and $\beta_s = (1 + \lambda(\beta - 1))$, which reduces the difference in the values of α and β to 0.1, further softening the potential during the scaling. Figures S16 and S17 confirm smooth scaling of the potential to zero and good overlap at all λ windows. We note that this scaling is not necessarily optimal, and further convergence improvements may be possible.

Table S7 further investigates the convergence of the aqueous and non-aqueous free energy calculations with the employed λ schedule for the LJ soft-core and DE-FF force fields. In

general, the variation across triplicate runs and convergence with the number of λ windows is slightly better for the LJ soft-core potential. However, all calculations are well converged even using just six evenly-spaced windows.

Finally, it has been previously noted that the soft-core LJ potential can lead to unwanted minima at intermediate alchemical states, in which two atoms become trapped at short distances from each other.[11] Figure S18 illustrates this situation for $\lambda = 0.5$. With the unmodified LJ potential, the two atoms are strongly repelled, but with the soft-core LJ potential there is a region at small r with small or zero force between the atoms. In contrast, the DE-FF potential remains repulsive at short range, but is softer than the unmodified LJ potential, which should lead to more robust free energy calculations.[11]

Table S7: Convergence of free energy results with number of λ windows. The free energy (kcal/mol) required to annihilate a molecule of ethanol in aqueous and non-aqueous (2-Methylpyridine) solvent is computed using 6, 11 and 16λ windows. The calculations are performed in triplicate, using a simulation length of 2 ns, and the standard deviation across the runs is reported in parentheses.

		6	11	16
aqueous	LJ soft-core	3.00 (0.12)	3.00 (0.05)	3.02 (0.05)
	DE-FF	3.71 (0.28)	3.66 (0.16)	3.65 (0.10)
non-aqueous	LJ soft-core	3.00 (0.09)	2.93 (0.04)	2.92 (0.07)
	DE-FF	3.38 (0.23)	3.36 (0.05)	3.41 (0.15)

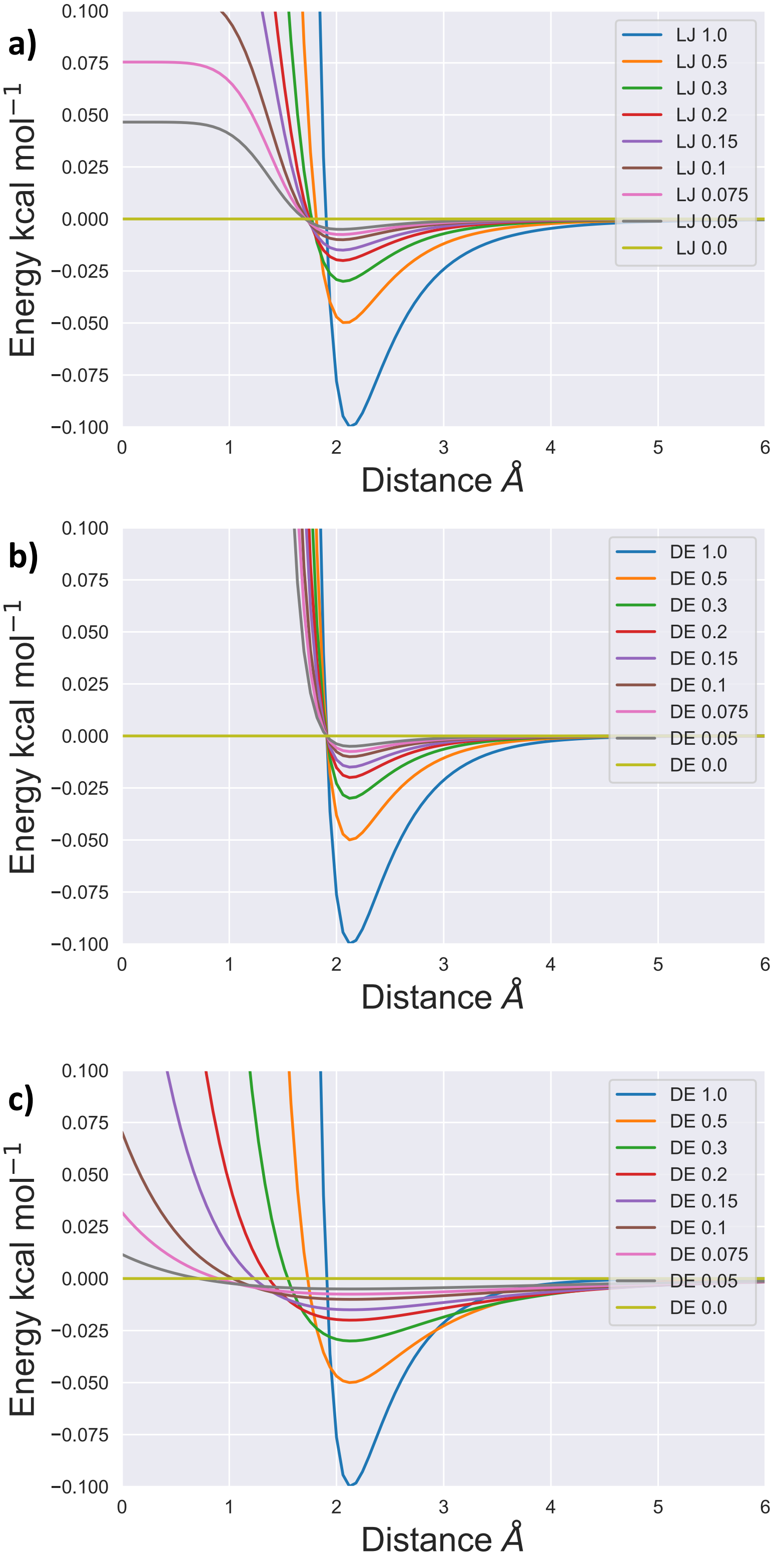

Figure S16: Variation of a) the LJ soft core potential, and two schedules of the DE-FF with the alchemical λ (using b) eq 4 and c) eq 5).

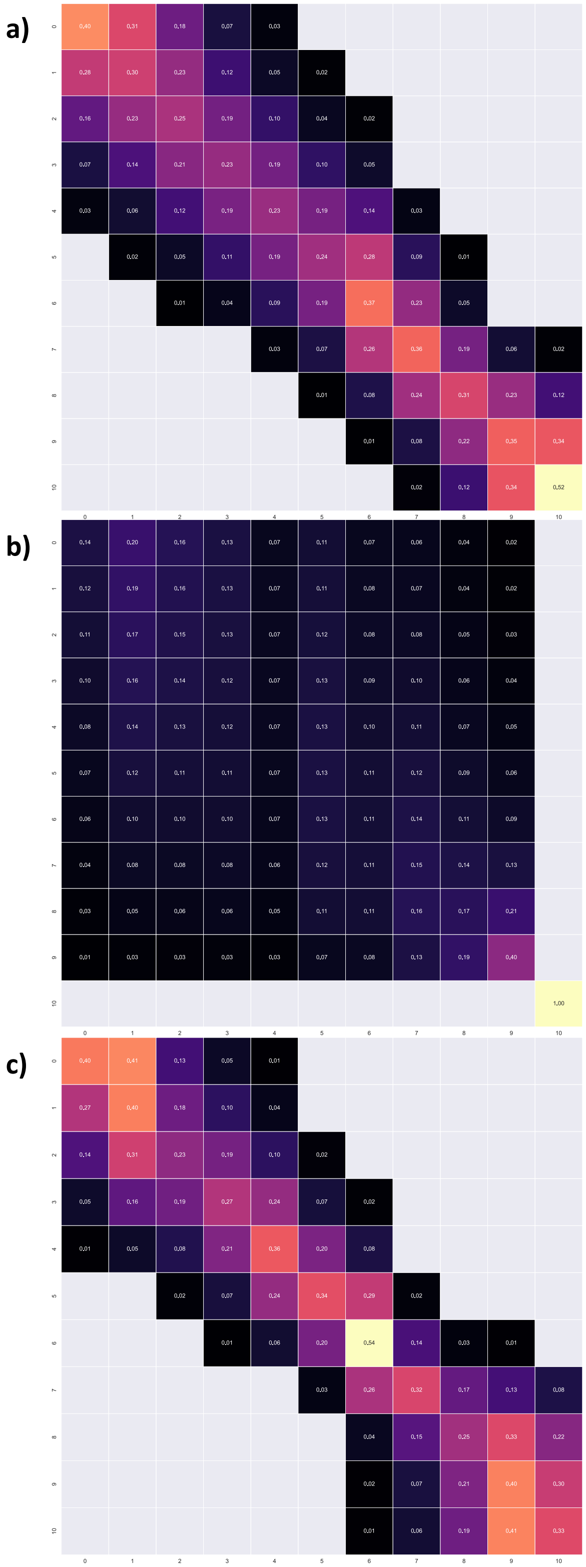

Figure S17: Overlap matrices extracted from non-aqueous free energy calculations of ethanol in 2-methylpyridine for 11 equally-spaced λ windows for the vdW annihilation stage. a) Sage shows good overlap as expected with the soft-core LJ potential. b) DE-FF shows poor overlap with the end state when using linear scaling due to the sudden disappearance of the potential at small λ. c) This is corrected using our scaled protocol (eq 5), which shows good overlap.

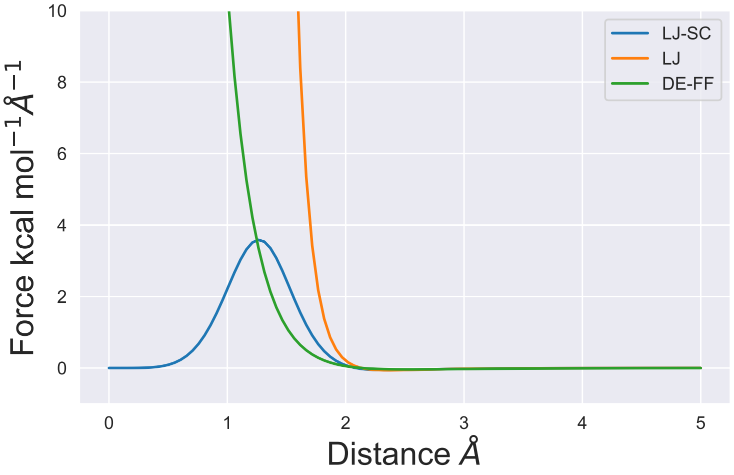

Figure S18: The DE non-bonded potential does not suffer from the additional minima which occasionally arise in the soft-core LJ potential.[11] Force curves for two atoms interacting at $\lambda = 0.5$ via the Lennard-Jones (LJ), soft-core LJ (LJ-SC) and double exponential (DE-FF) potentials only.

S5 Supplementary Computational Details

All simulations were run with OpenMM-7.6.0 on the cuda platform.[5] All liquid simulation boxes were created using PackMOL[12] via OpenFF-Evaluator. The systems were parameterised using the openff-toolkit-0.10.6 and smirnoff-plugins-0.0.2. ForceBalance-1.9.2 was used to optimise the non-bonded parameters via an interface to OpenFF-Evaluator-0.3.11.[13] A non-bonded cutoff distance of 9 Å was used in all liquid simulations and a switching function was applied to smoothly reduce the vdW potential to zero over the last 1 Å. The convergence of the solvation-free energy calculations with respect to the cutoff range was investigated to ensure the correct application of the long-range correction with the custom functional form (Figure S19). All statistical performance metrics are reported with 95% confidence intervals from 1000 iterations of bootstrapping with replacement.

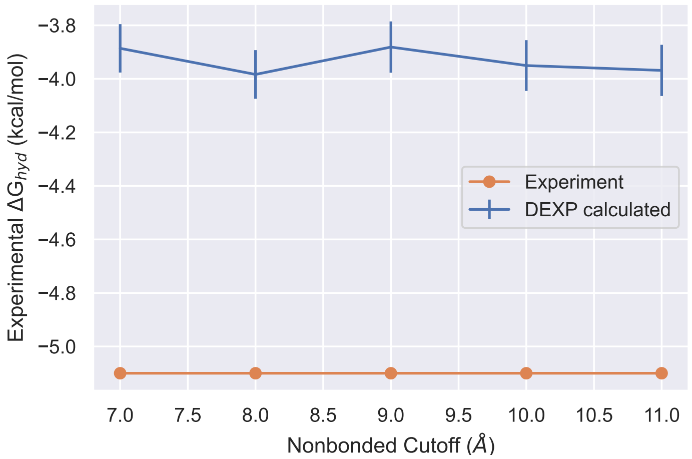

Figure S19: Dependence of the aqueous solvation free energy of methanol with non-bonded cut-off distance. Through the use of the long-ranged correction, the free energy is converged after 7 Å.

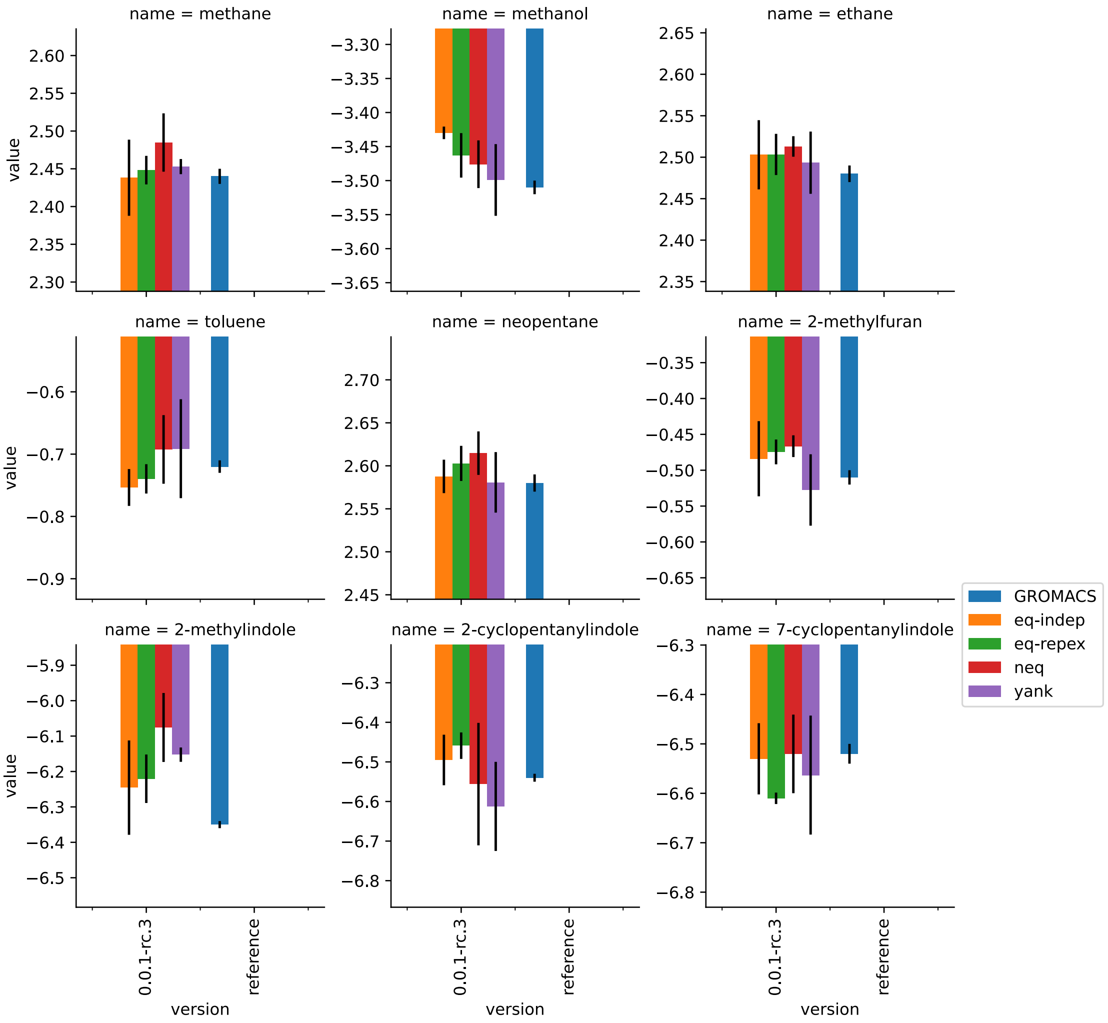

Figure S20: Regression tests for absolute hydration free energies in Absolv. Free energy changes are computed in triplicate in Absolv using non-equilibrium (neq) and equilibrium procedures, with independent replicas (eq-indep) and replica exchange (eq-repex). Also shown for comparison are the same quantities computed using the Yank[14] and Gromacs[15] software packages.

References

(1) Mohebifar, M.; Rowley, C. N. An efficient and accurate model for water with an improved non-bonded potential. *J. Chem. Phys.* **2020**, *153*, 134105.

(2) Hermann, J.; DiStasio, R. A. J.; Tkatchenko, A. First-Principles Models for van der Waals Interactions in Molecules and Materials: Concepts, Theory, and Applications. *Chemical Reviews* **2017**, *117*, 4714–4758.

(3) Tang, K. T.; Toennies, J. P. An improved simple model for the van der Waals potential based on universal damping functions for the dispersion coefficients. *J. Chem. Phys.* **1984**, *80*, 3726–3741.

(4) Man, V. H.; Wu, X.; He, X.; Xie, X.-Q.; Brooks, B. R.; Wang, J. Determination of van der Waals Parameters Using a Double Exponential Potential for Nonbonded Divalent Metal Cations in TIP3P Solvent. *J. Chem. Theory Comput.* **2021**, *17*, 1086–1097.

(5) Eastman, P.; Swails, J.; Chodera, J. D.; McGibbon, R. T.; Zhao, Y.; Beauchamp, K. A.; Wang, L. P.; Simmonett, A. C.; Harrigan, M. P.; Stern, C. D.; Wiewiora, R. P.; Brooks, B. R.; Pande, V. S. OpenMM 7: Rapid development of high performance algorithms for molecular dynamics. *PLoS Comput. Biol.* **2017**, *13*, 1–17.

(6) Wang, L.-P.; Martinez, T. J.; Pande, V. S. Building Force Fields: An Automatic, Systematic, and Reproducible Approach. *J. Phys. Chem. Lett.* **2014**, *5*, 1885–1891.

(7) Donchev, A. G.; Taube, A. G.; Decolvenaere, E.; Hargus, C.; McGibbon, R. T.; Law, K.-H.; Gregersen, B. A.; Li, J.-L.; Palmo, K.; Siva, K., et al. Quantum chemical benchmark databases of gold-standard dimer interaction energies. *Scientific data* **2021**, *8*, 55.

(8) Boothroyd, S. et al. Development and Benchmarking of Open Force Field 2.0.0: The Sage Small Molecule Force Field. *J. Chem. Theory Comput.* **2023**, *0*, null, PMID: 37167319.

(9) Mobley, D. L.; Guthrie, J. P. FreeSolv: a database of experimental and calculated hydration free energies, with input files. *J. Comput. Aid. Mol. Des.* **2014**, *28*, 711–720.

(10) Beutler, T. C.; Mark, A. E.; van Schaik, R. C.; Gerber, P. R.; van Gunsteren, W. F. Avoiding singularities and numerical instabilities in free energy calculations based on molecular simulations. *Chem. Phys. Lett.* **1994**, *222*, 529–539.

(11) Gapsys, V.; Seeliger, D.; de Groot, B. L. New Soft-Core Potential Function for Molecular Dynamics Based Alchemical Free Energy Calculations. *J. Chem. Theory Comput.* **2012**, *8*, 2373–2382.

(12) Martínez, L.; Andrade, R.; Birgin, E. G.; Martínez, J. M. PACKMOL: A package for building initial configurations for molecular dynamics simulations. *J. Comput. Chem.* **2009**, *30*, 2157–2164.

(13) Boothroyd, S.; Wang, L.-P.; Mobley, D. L.; Chodera, J. D.; Shirts, M. R. Open Force Field Evaluator: An Automated, Efficient, and Scalable Framework for the Estimation of Physical Properties from Molecular Simulation. *J. Chem. Theory Comput.* **2022**, *18*, 3566–3576, PMID: 35507313.

(14) Rizzi, A.; Grinaway, P. B.; Parton, D. L.; Shirts, M. R.; Wang, K.; Eastman, P.; Friedrichs, M.; Pande, V. S.; Branson, K.; Mobley, D. L.; Chodera, J. D. YANK: A GPU-accelerated platform for alchemical free energy calculations. `http://getyank.org`.

(15) Páll, S.; Zhmurov, A.; Bauer, P.; Abraham, M.; Lundborg, M.; Gray, A.; Hess, B.; Lindahl, E. Heterogeneous parallelization and acceleration of molecular dynamics simulations in GROMACS. *J. Chem. Phys.* **2020**, *153*, 134110.
